# A framework for handweaving robotic textiles with liquid crystal elastomer fibers

**DOI:** 10.1038/s41598-025-97835-1

**Published:** 2025-05-15

**Authors:** Sarah Nicita, James C. Weaver, Hiroshi Ishii, Jack Forman

**Affiliations:** 1 Tangible Media Group, MIT Media Lab, Cambridge, MA USA; 2 Harvard’s Wyss Institute for Biologically Inspired Engineering, MIT Department of Civil and Environmental Engineering, Cambridge, MA USA

**Keywords:** Materials for devices, Soft materials

## Abstract

Textile production methods present a rich set of strategies for developing materials with both form and function encoded at the fiber scale. Beyond simply acting as a static flexible barrier, the ability to incorporate environmentally responsible materials into fabric architectures significantly expands the textile design space by adding on-demand and programmable 3D structural morphing. To this end, liquid crystal elastomers (LCEs) are a promising candidate for enabling these reversible actuation behaviors in fabric-based constructs. Drawing on traditional textile manufacturing techniques and through a detailed exploration of the vast woven textile design space, we have demonstrated programmable and reversible curling, puffing, and in-plane shrinkage behaviors by embedding the functionality of LCE fibers into single and multi-layered woven structures. Predictable shifts in fabric structure directly influence the mechanical properties and the resulting form factor of the actuated textiles, which can in turn be effectively leveraged for the generation of multi-functional devices, enabling new directions for the engineering of flexible stimuli-responsive materials.

**Supplementary Information:**

The online version contains supplementary material available at 10.1038/s41598-025-97835-1.

## Introduction

Textiles are one of humankind’s earliest engineered technologies, with string abaci and woven baskets dating back millennia^1^. The versatility and adaptability of fabrics drive their central role in everyday life, with applications that range from clothing to shelter. As a promising class of materials for engineering applications, textiles couple a flexible, lightweight nature with the potential for programming physical properties across different length scales^2,3^. Transitioning from viewing textiles as substrates from which active components such as power supplies, actuators, and sensors are attached, fiber scientists and textile engineers have begun leveraging the rich history of textile construction methods to develop fabrics with integrated actuators and sensors^1,4–11^. When electronics are functionally integrated into specific textile architectures, hard components like circuits are recast as soft and flexible systems. With these advantages in mind, engineers have turned to textile-based materials for use in soft autonomous garments, flexible robotic systems, adaptive architectures, and biomedical therapeutics^1,3,12–15^. As material scientists continue to develop intelligent fibers that respond to an even wider range of environmental stimuli, there is an equally broadening design space for the incorporation of new functionalities into textile-based devices.

### Knitted and woven fabrics

**Fig. 1:**
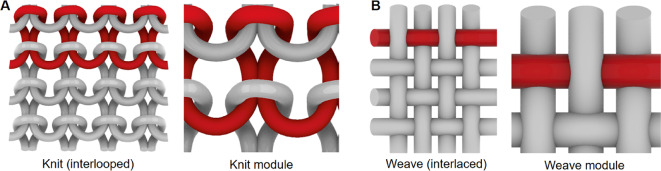
Knitted and woven fabrics. Low and high magnification views of a typical interlooped knit (**A**) and interlaced woven (**B**) textile. For each image, an individual fiber path is highlighted in red.

Knit and woven fabrics (Fig. 1) exhibit unique properties based on programmable variations in their structural hierarchy^2,16–18^, spanning from fiber to yarn, to fabric structure, and final fabric form. Fiber is formed into yarn, which is then transformed into varying fabric structures that govern the mechanical properties of the textile. Despite the widespread use of both manufacturing technologies in the garment industry, knits and wovens, due to their structural differences, exhibit unique sets of material properties in terms of durability and anisotropy^19,20^. Knitted textiles are composed of interlocking loops of yarn, resulting in a fabric with high elasticity and significant anisotropy. The continuous fiber used in knitting enables rapid production and mechanical flexibility, but the resulting fabrics exhibit reduced structural durability, as a single yarn break can cause the fabric to unravel. Woven textiles, by contrast, are composed of yarns interlaced at right angles, enabling mechanical properties such as high tensile strength and minimal stretch. While both knitting and weaving have unique levels of programmability, basic weaving allows for control over the simultaneous tuning of material properties running lengthwise and crosswise through the interlacing of two distinct elements (warp and weft), while knitting primarily allows for the material programming of loops running lengthwise. For this reason, the research presented here focuses on the design and manufacturing of woven fabrics.

**Fig. 2:**
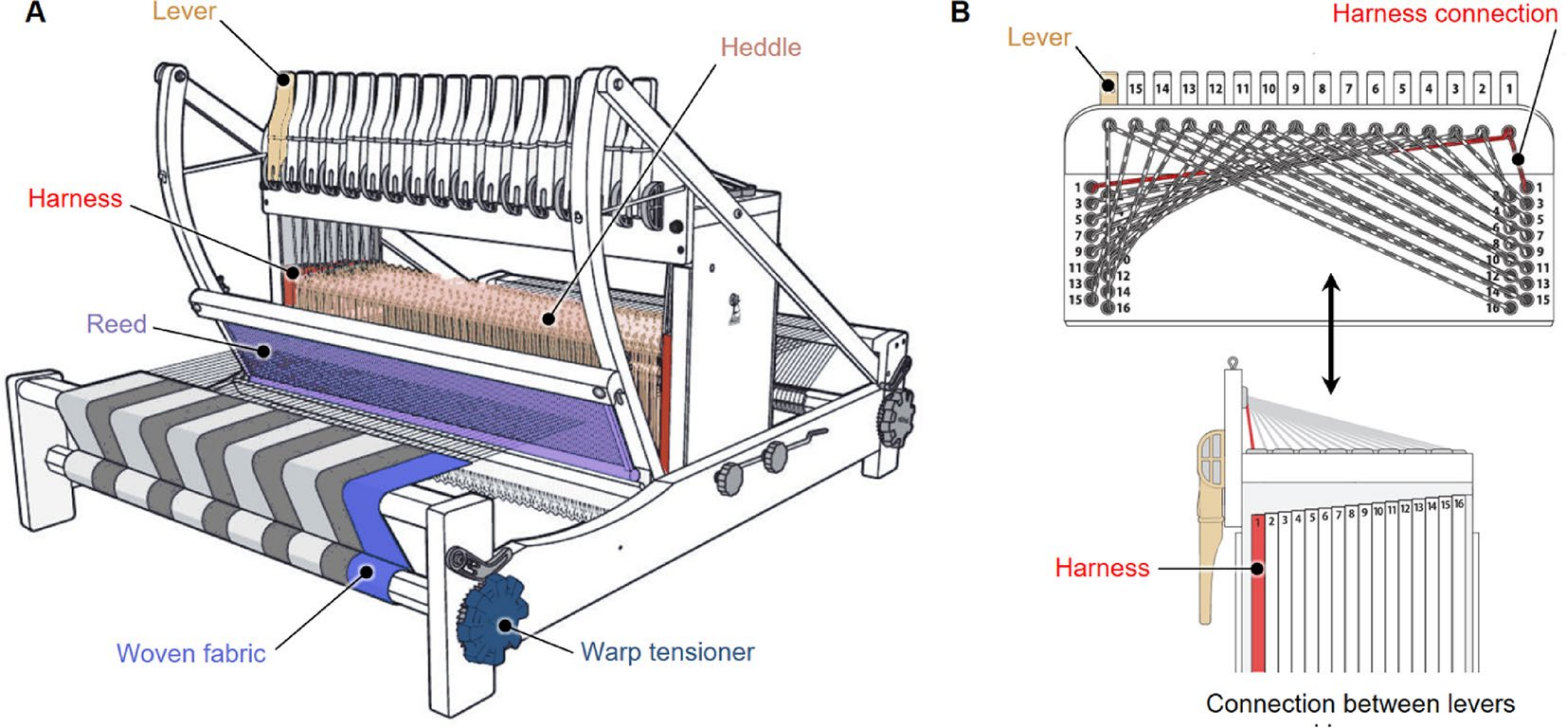
Functional schematic of a standard textile loom (**A**), and overview of the yarn lifting mechanisms used in creating woven geometries (**B**) In this diagram, the different components of the loom including the levers, heddles, harnesses, reed, warp tensioner, and woven fabric are labeled. Levers are attached to harnesses through string connections, enabling the controlled lifting and lowering of different warp threads. Each harness contains hundreds of heddles, and the warp threads are placed through these heddles in specific sequences. These warp threads are then manually threaded through the reed and tensioned along a beam at the front of the loom. A weft yarn is passed back and forth through a channel in the warp, which is created when different harnesses are lifted, creating the woven fabric over time.

**Fig. 3:**
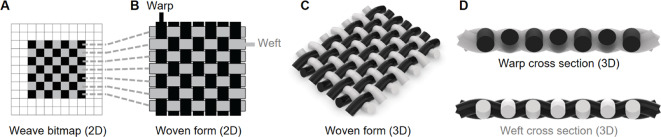
Diagram illustrating how bitmap patterns are encoded in a woven form. (**A**) a bitmap diagram of the relationship between warps and wefts in a woven draft, (**B**) a 2D illustration of a woven form, and (**C**) a 3D model of the resulting plain weave structure. (**D**) Models of warp and weft cross-sections illustrating how in a perfectly balanced weave structure, the warp and weft are the inverse of one another.

In the production of woven fabrics, warp yarns/fibers are tensioned vertically, and weft strings are perpendicularly passed over and under the warp to create different structures. These intersections are enabled by the threading of warp yarns through a sequence of heddles on the loom (Figs. 2, S1, S2), and variations in these sequences allow for the creation of interlaced fibers, known as weave structures (Fig. 3). Precise programmable control over the distribution of warp and weft fibers (which can be simply depicted using a bitmap-like notation, Fig. 3), therefore presents an opportunity for the creation of intelligent, multi-layered textiles through variations in yarn architecture by modulating properties such as yarn material, twists per inch, and cross-sectional organization^5,8,21–23^. Fabric architecture can be further altered by modifying the interlacing of yarns as structural patterns^17,18,24–27^. Beyond different patterns, weaving also permits the simultaneous and location-specific incorporation of different materials, thus allowing for tunable stiffness, transparency, conductivity, and color^26,28–30^. These differences in yarn properties, component placement, and patterning can result in a range of active and inactive behaviors and properties, thus defining the structural, haptic, and mechanical properties of a woven material.

Fiber or yarn tensioning during the weaving process play a critical role in determining the final shape of an active or inactive woven fabric, as warp and weft yarns can be individually tensioned to change the resulting woven structure through relaxation and differential shrinkage. At the fiber level, materials like elastics can carry tension due to their ability to stretch, leading to significant structural change when a woven structure is removed from the loom and subsequently relaxes; yarns adjust and settle into a different configuration in the absence of warp and weft tension^31^. Furthermore, differential shrinkage can take place if the weave is post-processed in a manner that transforms fiber material properties; for example, when wool fibers are exposed to water and heat, scales along the fibers interlock to create an irreversibly denser and tighter structure^32^. Furthermore, environmental factors, such as moisture absorption, can also be a catalyst for fiber relaxing or swelling, resulting in the induction of intentional or unintentional fabric-wide dimensional or geometric changes^8^.

### Actuated textiles

Actuated textiles are fabrics that are engineered to respond to stimuli and exhibit dynamic behavior^1,4,16,19^, and are created by integrating actuatable materials into traditional knit and woven textile structures^31,33,34^. The advantage of these material technologies lie in their familiar form factor, flexibility, durability, and lightweight construction, and previous work has leveraged the programmable nature of knit and woven structures to develop responsive fabrics for thermoregulation, haptics, therapeutic compression, and grasping applications^1,23,35–38^. Programmable geometric concepts such as kirigami and origami have also been leveraged for the production of knitted soft robotics with desired self-folding and auxetic kinematic behaviours^1^.

To achieve actuation in these textile-based materials, bladder-based fluidic actuators, cable-driven actuators, and shape-changing material-based actuators have recently been employed to create knit and woven textile architectures that change shape, stiffness, and/or conductivity in response to external forces such as heat, pressure, moisture, and electric currents^23,39,40^. For example, external or internal liquid or air sources can be employed to expand textile-covered bladder-based fluidic actuators to induce a shape change^1^, and variable stiffness can be achieved through graduated pressure levels. Within these contexts, custom textile patterns with directionally specific flexibility have also been successfully leveraged to create devices that exhibit a range of motions, including bending, contraction, and extension^40–43^. In an alternative approach, cable-driven actuators use external mechanical linkages to apply force to the textile^35^. While they can be used to create morphable textile constructs, most of these actuators are incompatible with traditional knitting or weaving hardware, and as such, they cannot be co-knitted or woven into a textile during fabrication, and are usually added in a secondary manufacturing process, or slid into a pre-formed void in the growing textile. To help address these limitations, the recent emergence of engineered morphing fibers with reversible extensile and contractile behaviours presents a growing design space for active woven textiles^17–19^, bridging previous work on engineered weave structures and novel fiber-based mechanisms.

### Environmentally responsive yarns

Active yarns are composed of shape-changing materials that can enable specific functions within a fabric structure. Within this larger category of multi-functional yarns, environmentally responsive yarns represent one such subclass and include those consisting of fluid-filled tubes (fluidic actuators), shape memory alloys (SMAs), and liquid crystal elastomers (LCE)^1,42,44–47^. Fluidic active yarn actuators can respond to changes in ambient pressure, temperature variations, moisture, and chemical gradients, and have been used for wearable applications, including medical textiles, and assistive devices^23,40,48^. Despite this diverse application space, the presence of an internal liquid channel within the constituent fibers adds an additional step to the fiber manufacturing process and somewhat limits the weave geometry design space due to problems associated with compression and kink formation within the tubular components^23,40^. One approach to resolving these design limitations is through the use of SMAs. The unique behavior of SMAs such as NiTi alloys rely on the cooling-induced phase transformation from austenite (a higher-temperature phase) to martensite (a lower-temperature phase), and linear actuation can be generated by preprogramming the material at high temperatures and then cooling the material to its martensite phase^49^. When reheated, the material will “remember” its programmed shape in the austenite phase, and repeated actuation is possible through applied heating and cooling, which can be controlled through an applied current. Representing one of the most developed fields in active textile materials, SMAs have been sewn, interlaced, knit, and woven to create compression garments and custom-fit wearables^50^.

### Liquid crystal elastomer (LCE)-based actuators

**Fig. 4:**
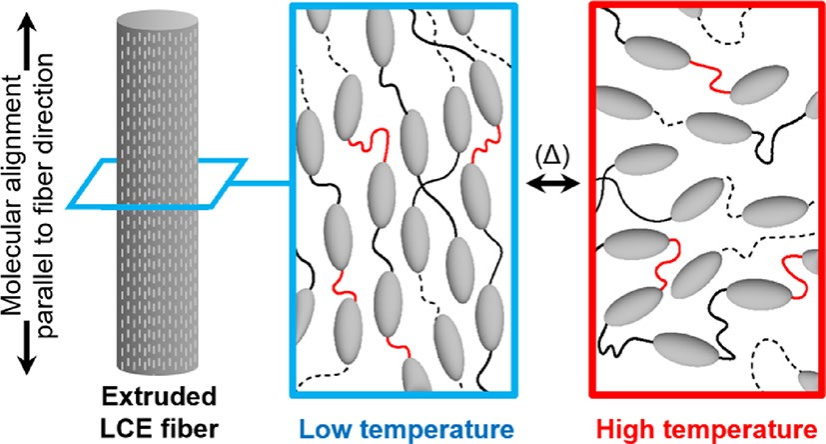
Molecular phase-change in a single extruded LCE fiber at low and high temperatures.

In contrast to SMA wires, which exhibit relatively low levels of linear extensibility and contractibility, LCE fibers (Fig. 4) can exhibit length changes up to 50% when exposed to external stimuli^51,52^. Shape-changing and elastic properties are enabled through the integration of long, flexible, cross-linked polymer chains with liquid crystal molecules (mesogens). The mesogens align themselves in specific orientations based on external conditions, and when these stimuli shift, they undergo a phase transition that alters molecular alignment, leading to macroscopic deformation in the polymer network. Due to these unique properties, LCE actuators have often been described as “artificial muscles”, and can be modified to respond and change shape in response to a wide range of environmental stimuli such as heat, electric fields, magnetic fields, and light, by simply varying their chemistry and their additives^16,52,53^. Furthermore, the versatility, biocompatibility, and efficiency of LCE actuators have allowed for advances in robotic and biocompatible smart textile-based applications^51,53,54^.

Based on this unique combination of desirable properties, LCE fibers represent a promising actuation technology due to their ease of integration into fabric substrates for pre-programmed behaviors such as stretching, bending, shrinking, and curling^38,52^. LCE fiber actuation is silent, rapid, and reversible, and as such, these properties have been leveraged to create intelligent micromachines, artificial muscles for exoskeletons, and active, responsive textiles^1,13,23,41,47,48^. Furthermore, recent advances in the development of continuous spinning technologies have allowed for the creation of LCE fibers that can be extruded at the scale of hundreds of meters, opening new opportunities for the production of large-scale, environmentally-responsive textile constructs^1,9,16^.

While existing research on digital simulations of shape-changing fabrics has permitted the exploration of new design spaces for active textiles^55^, the ease of LCE fiber manufacturing and its compatibility with standard knitting hardware presents new opportunities for the production and study of fully actuatable tangible textile prototypes. While previous efforts in this field have investigated morphing behaviors in knitted geometries composed entirely of LCE fibers^20^, a comprehensive understanding of how the fractional incorporation of LCE fibers into technologically relevant woven fabric architectures has remained largely unexplored. To bridge this knowledge gap, here we describe how weave geometry influences the applied forces and deformation modes of heat-response LCE-containing fabrics. Our process centers on combining weaving knowledge and programmable transformation in the design process, blending diverse materials and techniques.

## Results

**Fig. 5:**
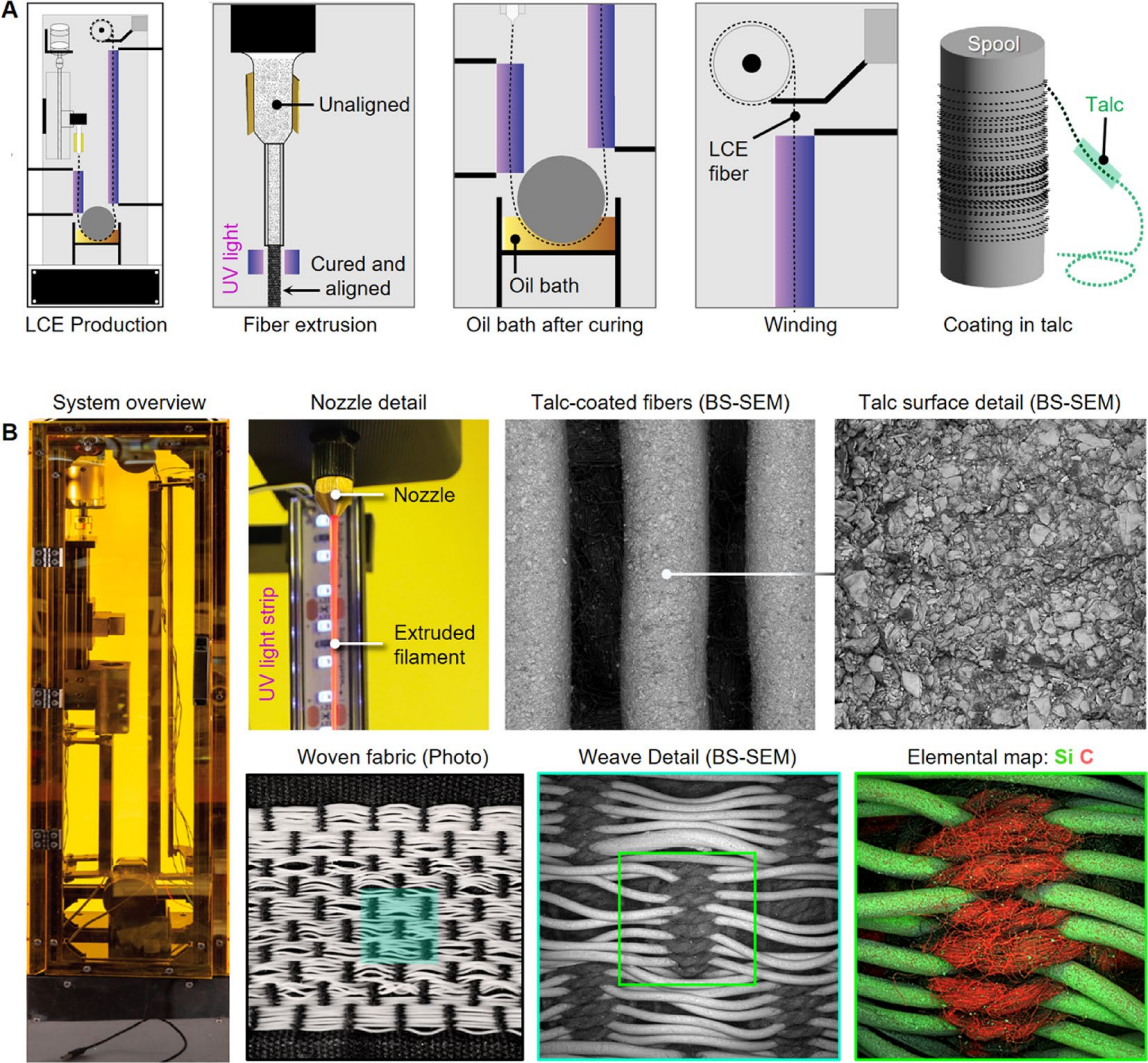
LCE fiber and fabric production. (**A**) Diagram of our LCE fiber manufacturing process, which includes fiber extrusion, winding, and coating. (**B**) A system overview, highlighting the UV-cured wet extrusion setup, talc-coated fibers with surface details (back-scattered scanning electron micrographs), and elemental mapping from a region of a woven fabric. The silicon (Si) signal comes from its high content in the talc mineral coating: Mg_3_Si_4_O_10_(OH)_2_. For scale, all of the LCE fibers each measure 1 mm in diameter.

The LCE fiber production and collection processes employed in the present study were modeled after those reported previously^43,56^. Specifically, this strategy begins by continuously extruding LCE fibers through a brass nozzle with an internal bore diameter of 1 mm. The resulting fibers were subsequently cured mid-air by passing them under an array of UV LEDs and then spooled onto a roll. An oil bath was used mid-cure to create a barrier between adjacent fibers during initial coiling to prevent the fibers from sticking to one another during the spooling process. LCE fibers were lastly coated in talc powder after curing to increase fiber longevity and reduce fiber-fiber friction during the weaving process (Fig. 5). Beyond these advantages, the talc coating offers an additional unintended benefit, in that it increases the average electron density of the fibers, making for their clear identification during backscattered scanning electron microscopy and elemental mapping-based imaging studies (Fig. 5). For all of the experiments described herein, 1 mm diameter LCE fibers were employed, and their measured mechanical properties were consistent with those reported previously^16,56^. Despite the great potential for leveraging high-speed automated weaving processes for the production of novel fabric architectures, for the work presented here, we chose to employ handweaving, which is highly customizable and permits strict control over fabric tension, making it beneficial for small-scale prototyping with new materials.

**Fig. 6:**
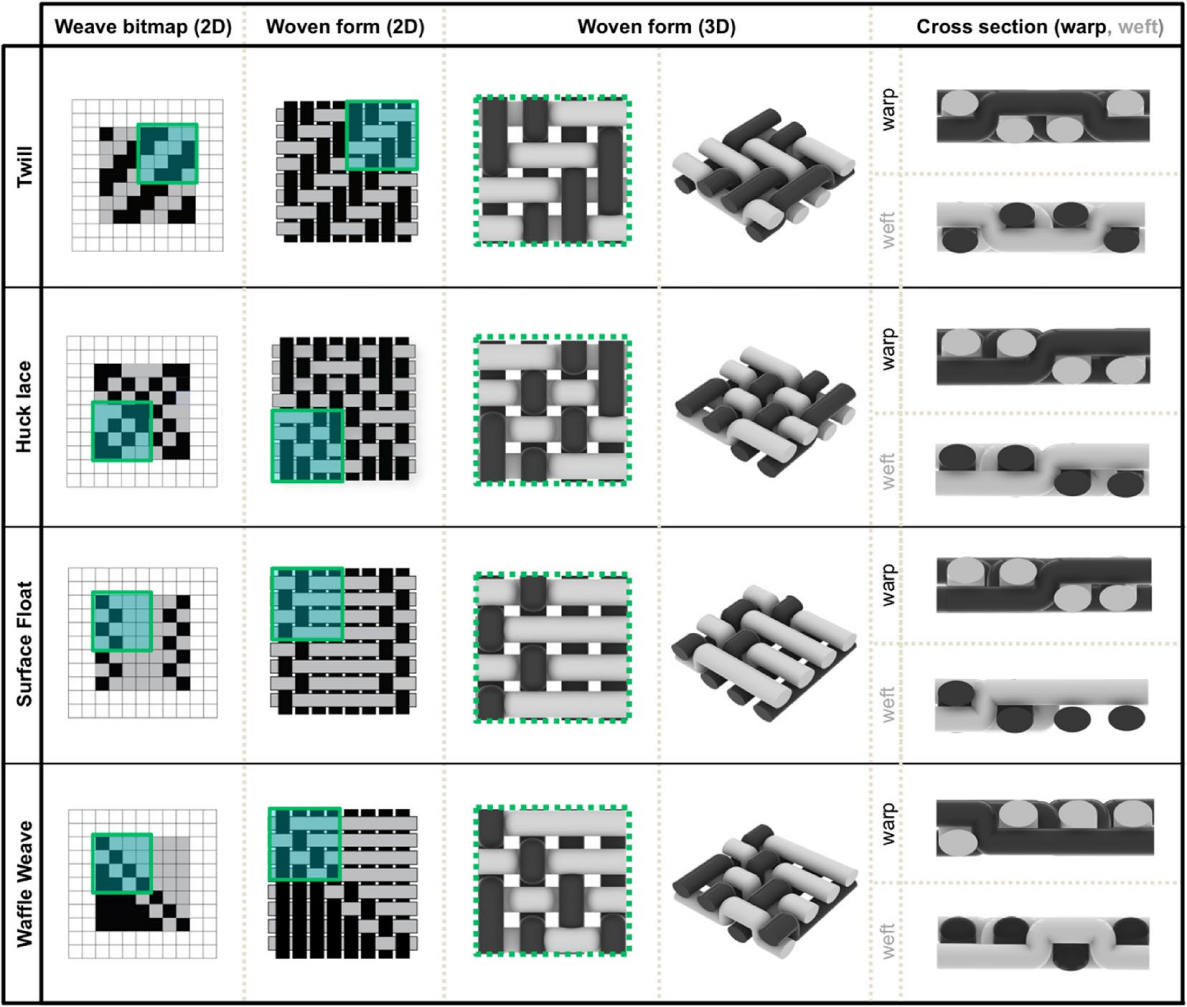
Diagrams illustrating the woven bitmaps, 2D woven forms, 3D woven forms, and warp/weft cross sections for a series of single-layer fabrics. In each row, the different highlighted boxes track a specific area of interest from one figure panel to the next.

**Fig. 7:**
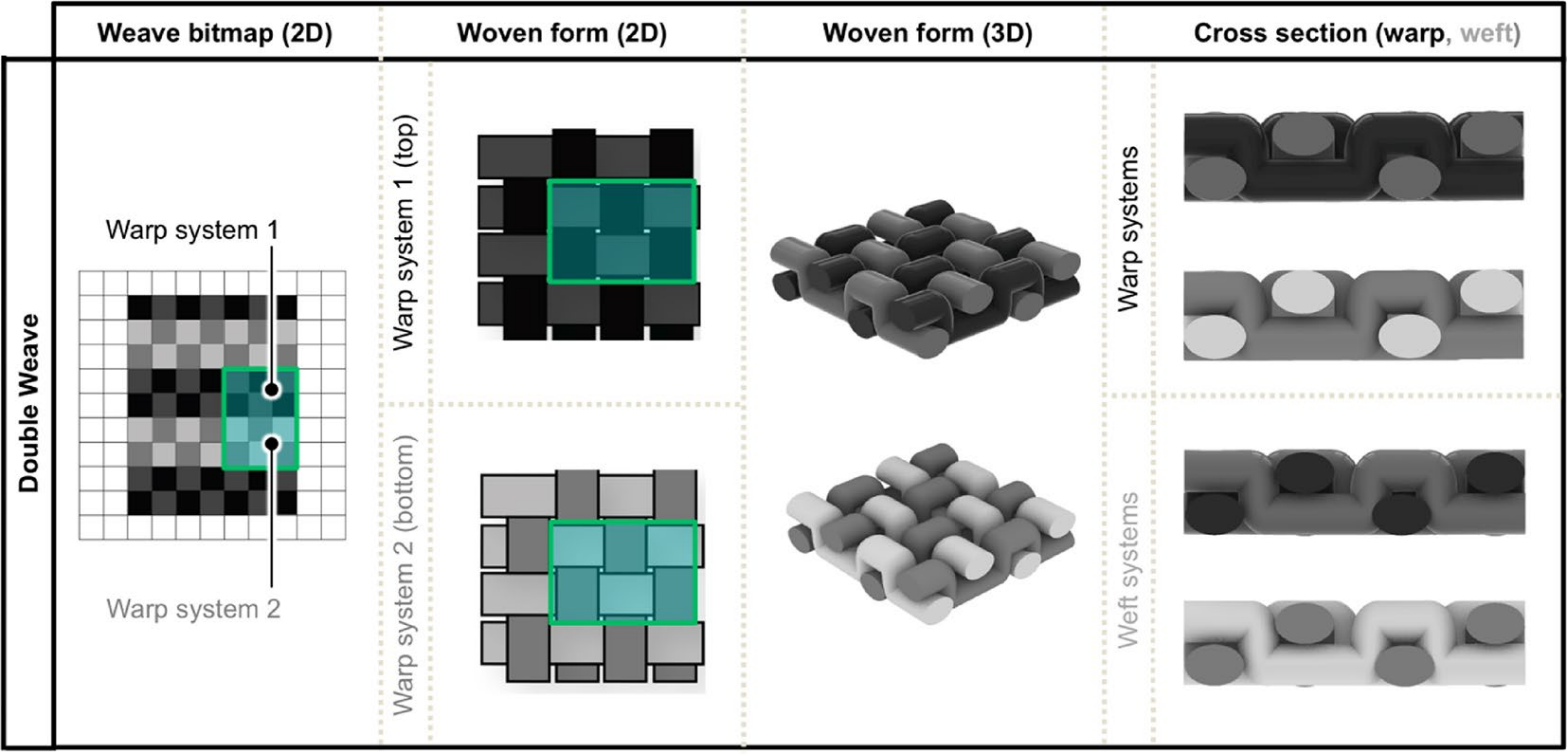
Diagrams illustrating the woven bitmaps, 2D woven forms, 3D woven forms, and warp/weft cross sections for a series of double-layer fabrics. In each row, the different highlighted boxes track a specific area of interest from one figure panel to the next.

To explore the actuatable design space, we incorporated our LCE fibers into a broad range of commercially relevant woven textile architectures guided by a hierarchy in the number of fabric layers (Figs. 6 and 7, S3, S4). Textile layers are defined by the number of warp systems that are used to create distinct fabric planes, resulting in variable fabric strength, flexibility, and topology^5^. While there are potentially thousands of weave patterns for single and multi-layered fabrics, this work focused on some of the most commonly utilized commercial patterns. The different fabric layers were created by manipulating threading techniques, warp and weft ends per inch, yarn tension, and tie-downs within each woven structure.

**Fig. 8:**
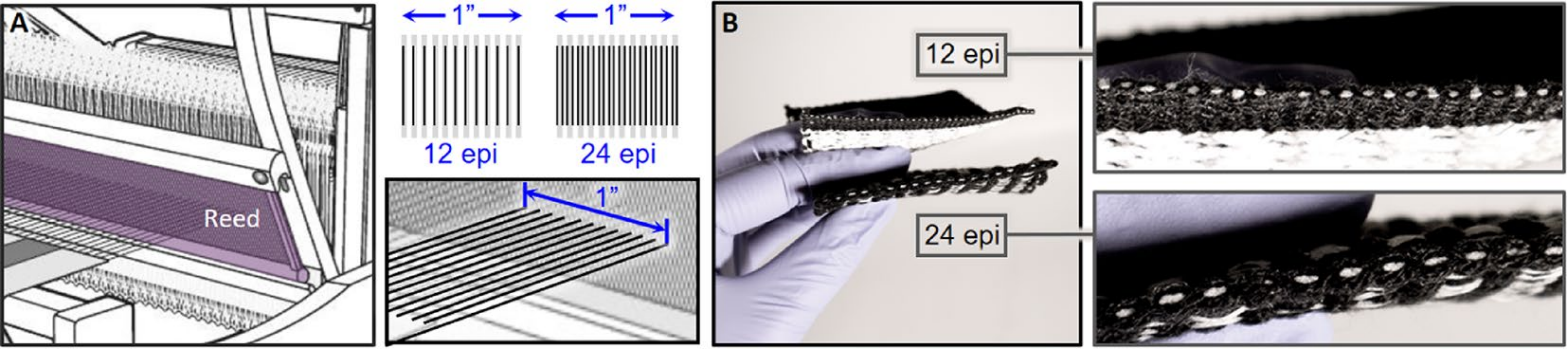
Diagram of how ends per inch is determined through warp structure (**A**) and two textile forms with 12 and 24 ends per inch (**B**).

Weaving parameters like ends per inch (EPI) and picks per inch (PPI) are important to consider during the design process, as they directly impact fabric density, texture, and structural stability. EPI refers to the density of yarns within one inch of a fabric warp; multi-layered fabrics have a higher count of warp ends per inch, and these ends are separated to weave many layers simultaneously. PPI refers to the density of weft yarns in one inch of the fabric width. A tightly packed fabric will have a higher PPI, while a more permeable fabric might have a lower PPI. In Fig. 8, the two samples shown have the same PPI and varying EPI based on the number of structural layers.

**Fig. 9:**
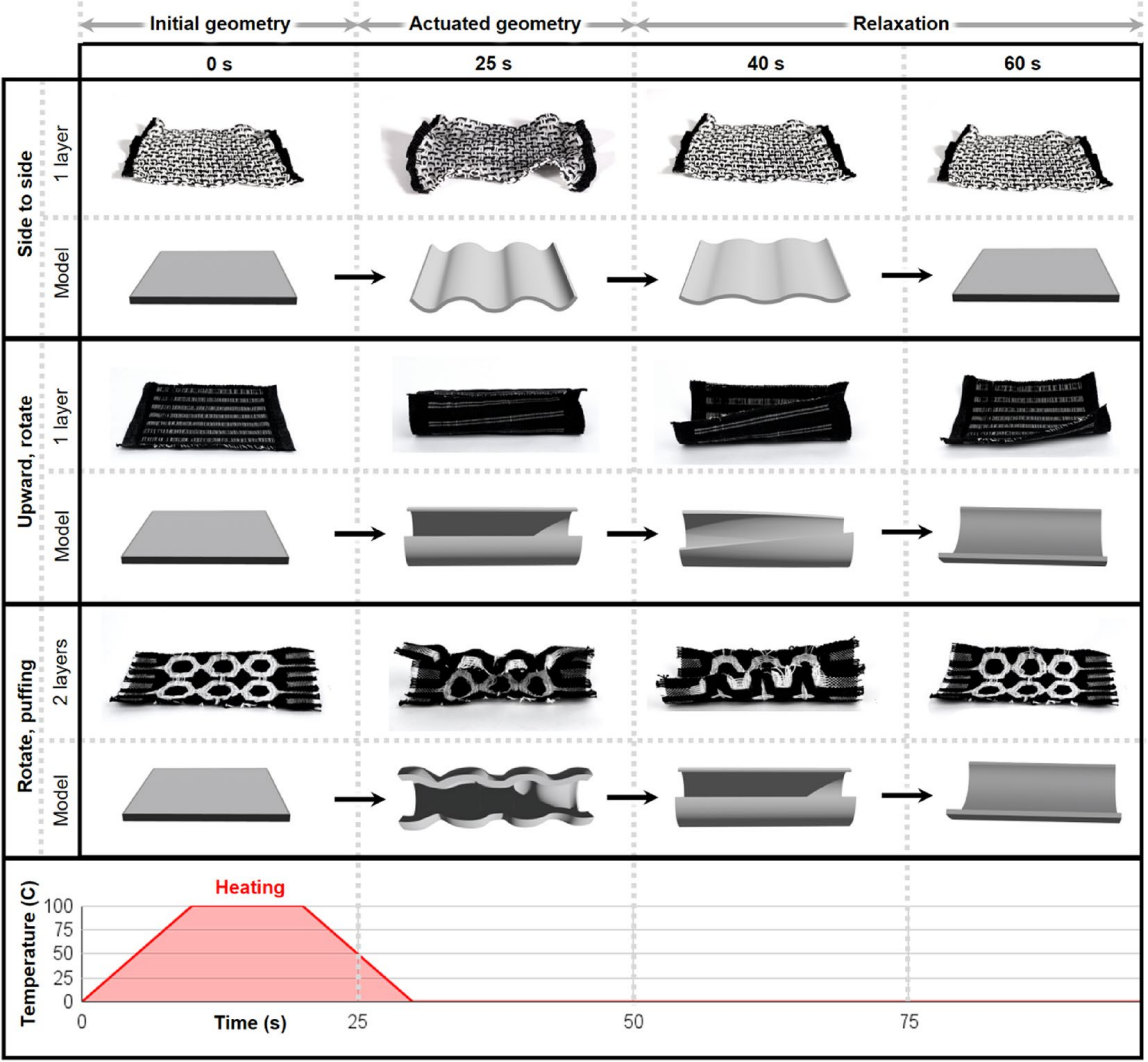
Diagrams illustrating basic actuation modes when subjected to external heating for 30 s (side to side, upward, rotational, and puffing movements) for single and double-layered fabrics over time.

The weave structures employed in the present study can be divided into two major classes based on their warp and weft density: single-layer fabrics and double-layer fabrics (Fig. 9). A detailed description of each weave structure listed in Fig. 9 can be found in Figures S3 and S4. After exposure to an external heat source, these different weave patterns resulted in predictable morphing behaviors.

The most straightforward single-layered fabric is the plain weave, which is commonly used to create functional fabrics such as sensors and memory units^4^. Other single-layer fabrics include, but are not limited to, twills, inlay patterns, varied float structures, and gauze fabrics. In the present study, we integrated LCE fibers into gauze-like single-layer fabrics such as spaced plain weave and mock leno, as well as denser single-layered geometries such as Swedish lace and waffle weave structures (see Figures SI3, SI4, and the methods section for details on these different textile architectures).

Multi-layer fabrics are composed of a warp that creates two different structural wefts and can be composed of a wide range of different yarn types, thus enabling diverse structural hierarchies^4^. A double-layer structure allows for the creation of pockets, channels, and multidimensional forms, and is a common design strategy seen in the e-textile literature^57^. The interplay between the two layers allows for the creation of textiles with distinct material properties: the thickness of a double weave depends on the number of tie-downs, or moments of intersection between the two layers^58^. To understand the interplay between these different parameters, the present study also investigated the actuation behaviors of fabrics with double-deflected wefts and double wovens with tie-downs and floats (see Figures S3, S4, and the methods section for details on these different textile architectures).

**Fig. 10:**
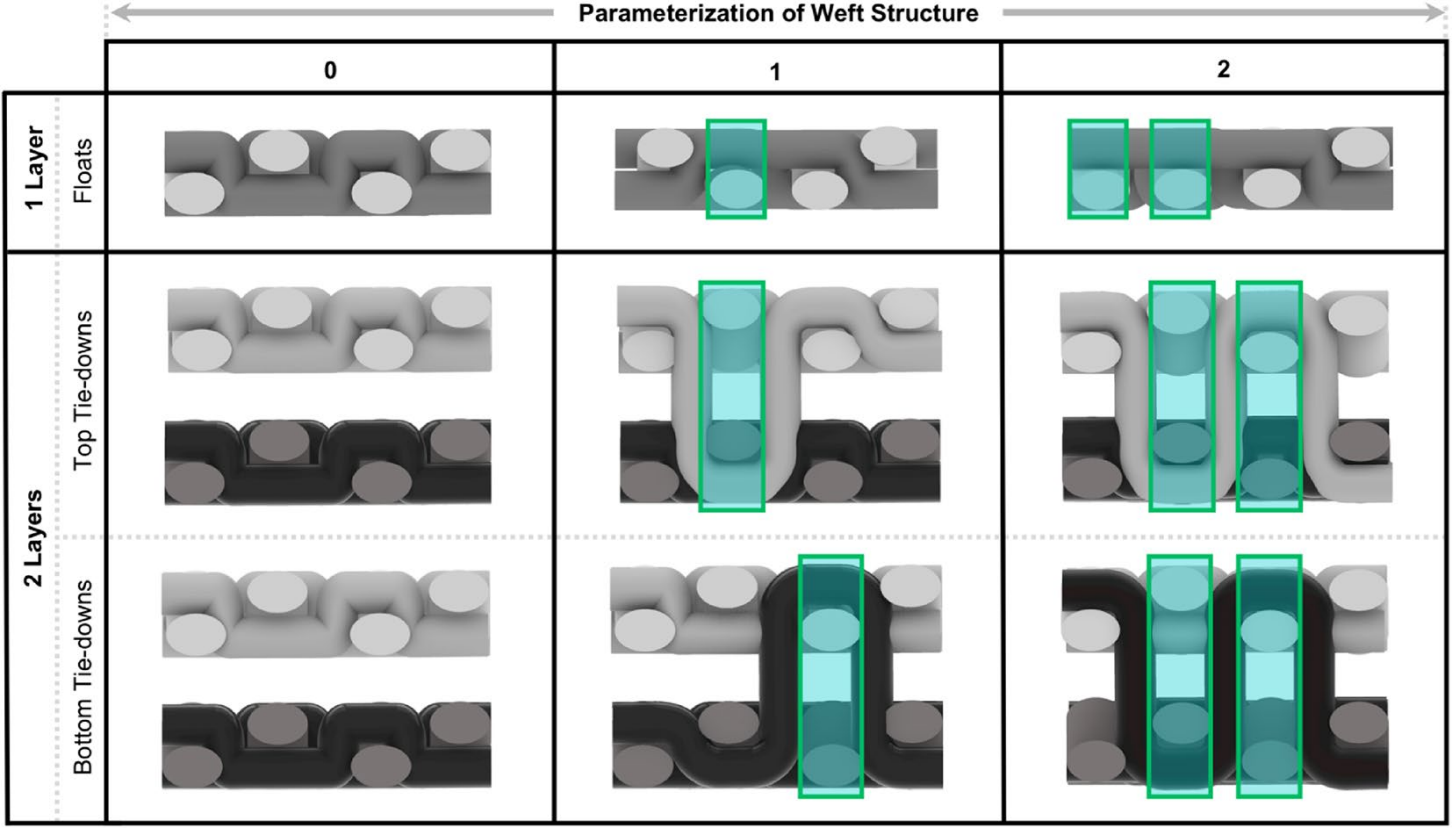
Diagrams illustrating different approaches to parameterizing weft structure for single and double-layer fabrics. The boxed regions denote locations along the woven textile where the structure is altered. For single-layer fabrics, this represents changes in the number of floats along the fabric’s surface. For double-layer fabrics, the boxes highlight the number of interactions between the two fabric layers.

A comprehensive understanding of actuation behavior is critical for designing, controlling, and operating responsive fabrics with specific performance characteristics, and for simplicity in the present study, the direction of motion was used to describe these behaviors. At the fabric level, tension is reduced through floats and introduced through tie-downs in the warp and weft matrix (Fig. 10). Tie-downs secure warp ends and are critical for maintaining uniform stability in a woven fabric.

**Fig. 11:**
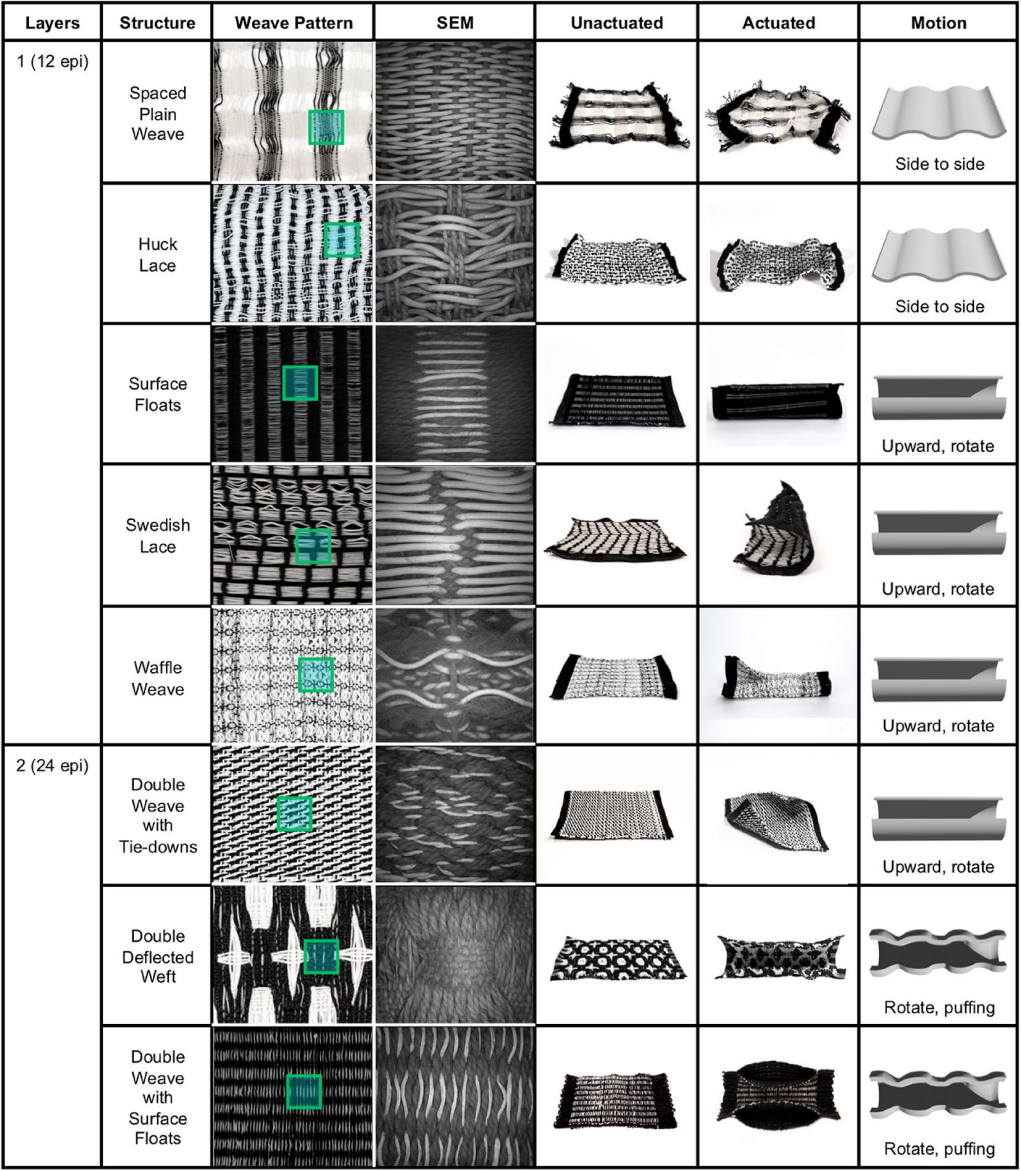
Overview of textile design space and associated heating-induced actuation motions. All SEM images measure 2 cm in width.

When designing with a shape-changing weft material, tie-downs may introduce friction between active and inactive fibers, resulting in different actuation behaviors. The intentional placement of different tie-downs within a textile structure can thus be leveraged for the parameterized control of the overall ranges of programmable motion. As noted in Fig. 11, uniform, consistent tie-downs with minimal floats on the front of the fabric result in side-to-side deformations (present in structures such as twills). Fewer tie-downs result in floats along the face and/or back of the fabric and allow for pronounced actuation behaviors such as curling (present in huck lace, waffle weave) and puffing (double-deflected weft, and multi-layered structures). Single-layer fabrics tend to curl upward, and contract (in a side-to-side fashion) based on the number of floats on the face or back of the fabric structure, while multi-layered fabrics tend to rotate and contract on more than one fabric plane, allowing for additional actuation behaviors such as expanding and puffing (Fig. 11).

**Fig. 12:**
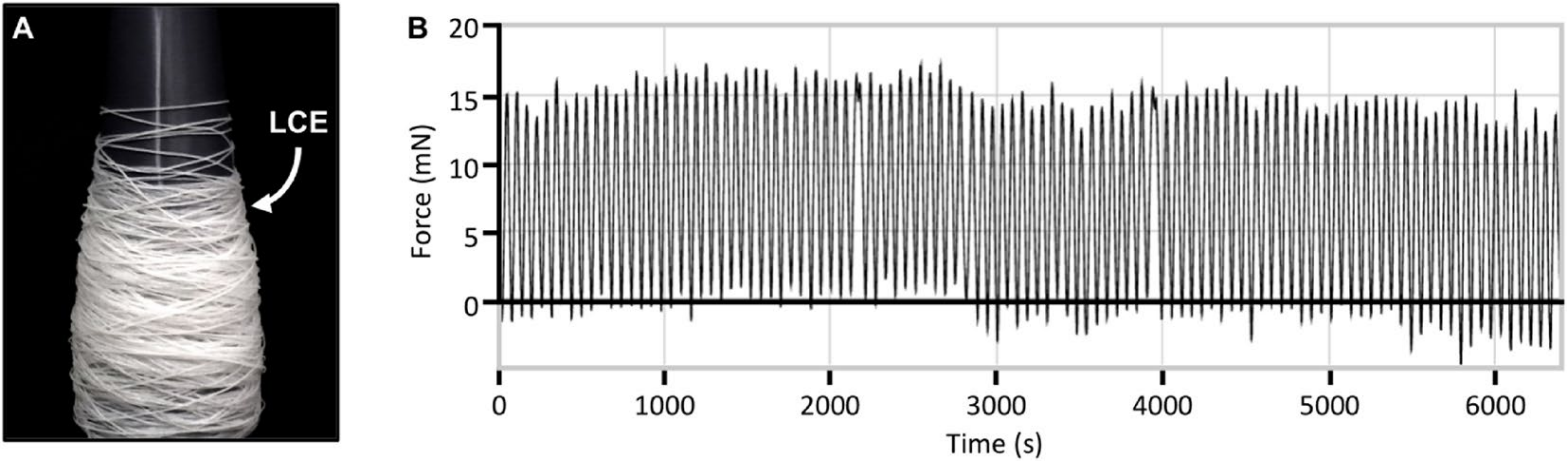
Actuation performance consistency of a single 1 mm-diameter LCE fiber over time. (**A**) roll of the extruded LCE fiber, and (**B**) a plot of contraction force as a function of time over 100 heating/cooling cycles.

Beyond these initial studies which provided a qualitative understanding of the actuation behaviors of the different woven architectures, our subsequent investigations were primarily focused on directly assessing their specific performance metrics. To quantitatively evaluate the properties of these LCE fibers as functional actuators, we first investigated the long term durability of a single 1 mm-diameter LCE fiber through approximately 100 heating and cooling cycles (Fig. 12). Throughout these measurements, minimal performance degradation was observed, which corroborated previously reported findings with similar material systems^16,43,51,52,54,56^.

**Fig. 13:**
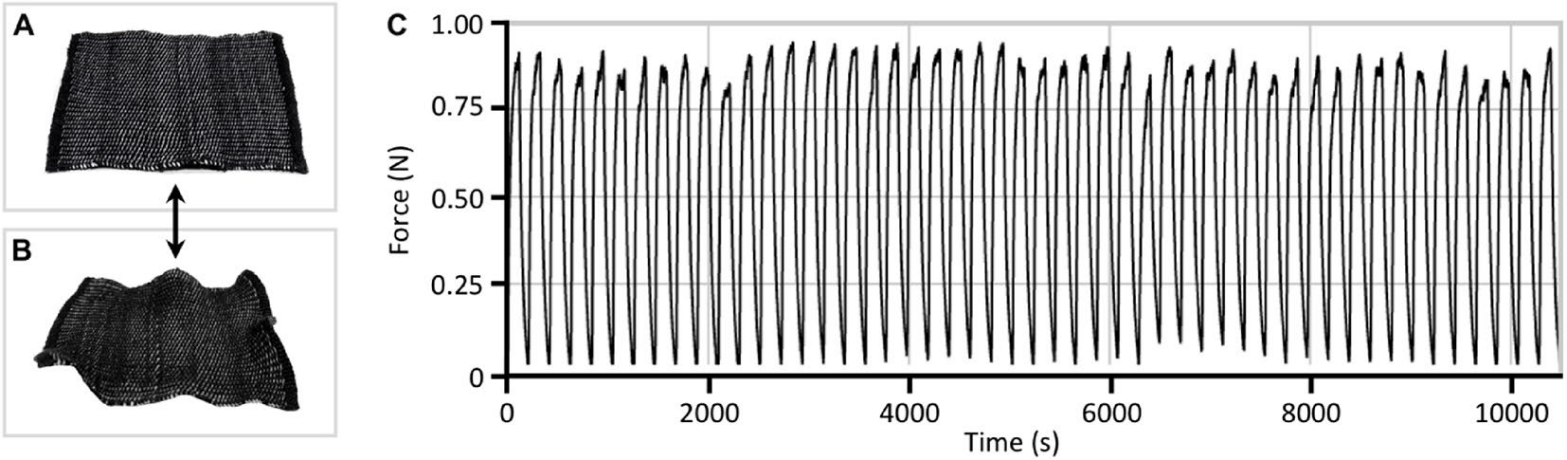
Actuation performance consistency of a twill sample over 50 heating/cooling cycles. (**A**) unactuated twill sample, (**B**) actuated twill sample, (**C**) plot of contraction force as a function of time over 50 heating/cooling cycles.

Expanding these measurements to the fabric scale, the mechanical performance of a twill sample was assessed through multiple heating/cooling cycles (Fig. 13). Corroborating the findings of previous studies^16,44,45,51,59–61^, minimal performance degradation was observed across 50 heating/cooling cycles, and when actuated, these textile samples demonstrated a maximum contraction force of approximately 0.9 N.

**Fig. 14:**
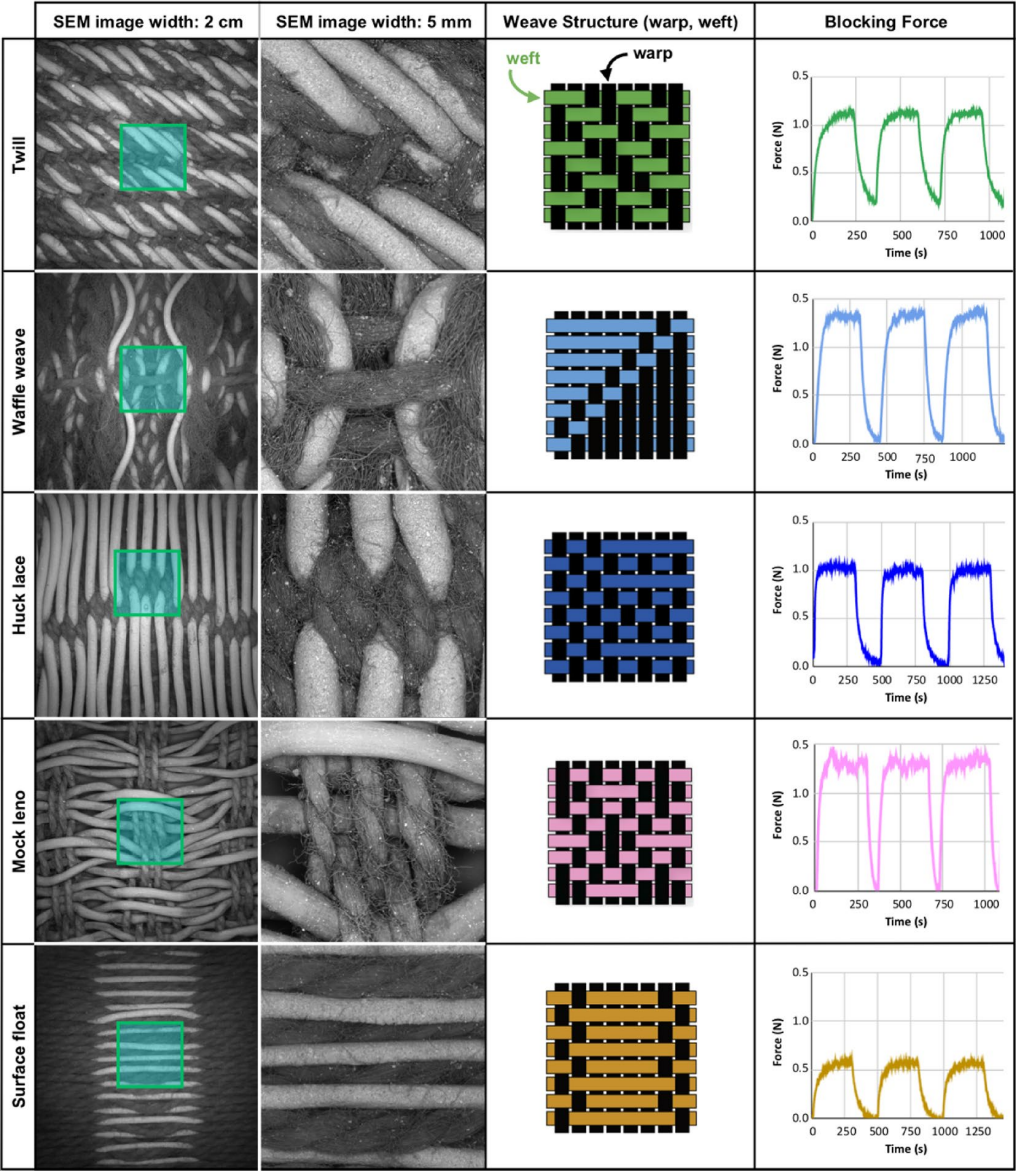
Overview of blocking force measurements for single-layer fabrics. Vertical columns detail specific weave structures (referenced in methods, and Figures S3, S4). Horizontal rows show low and high magnification SEM images of the fabric’s surface, drawings of fabric structure with LCE placement highlighted in different colors, and the actuated blocking force of each fabric structure over three heating/cooling cycles. Due to the inherently higher electron density of the LCE fibers (because of their talc coatings), these (brighter) fibers are clearly visible in the back-scattered SEM images.

**Fig. 15:**
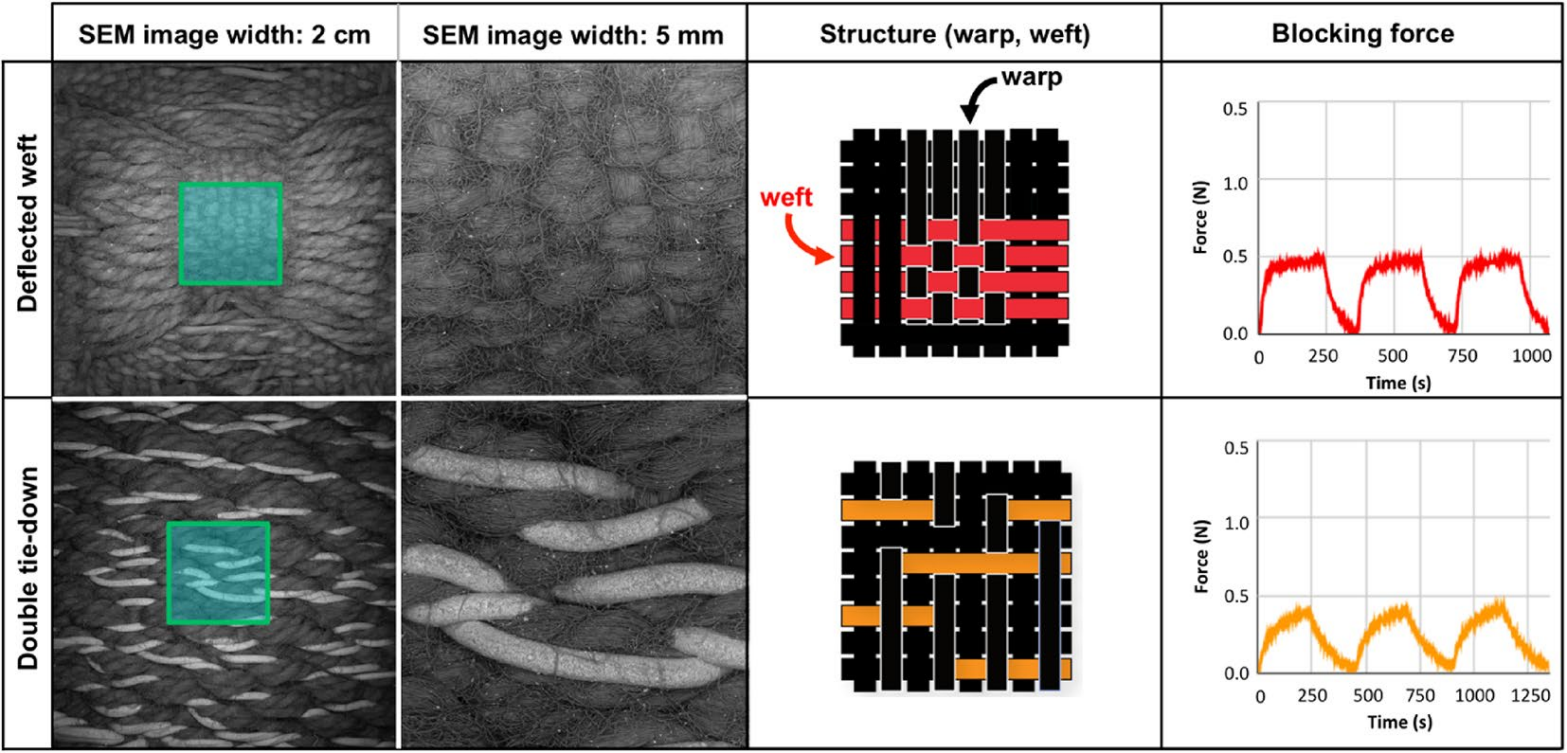
Overview of blocking force measurements for double-layered fabrics. Vertical columns detail specific weave structures (referenced in methods, and Figures S3, S4). Horizontal rows show low and high magnification SEM images of the fabric’s surface, drawings of fabric structure with LCE placement highlighted in different colors, and the actuated blocking force of each fabric structure over three heating/cooling cycles.

To understand how local interactions between weft and warp yarns in the textile matrix influenced blocking force (the maximum force generated by a powered actuator held at full elongation) when actuated, a series of mechanical tests on the different fabricated samples were performed using a Cellscale Univert S mechanical testing device equipped with a 1 N load cell (Figs. 14 and 15, and summarized in S5). During these tests, the fabric samples were placed 15 cm away from a heat gun that blew 160 °C hot air on them for 3-minute intervals with 2–3 min cooling times between heating cycles. For all of these tests, the fabric surface temperatures were ca. 65 °C (as measured with a thermal camera). The variability in cool-down time from sample to sample was influenced by the thermal properties of the woven fabric and was adjusted on a sample-by-sample basis (the heating cycle was initiated once the measured force returned to pre-testing values). Subtle differences in blocking force that related to the progressive increase in the amount of interaction between warp and weft yarns were observed across all of the samples. These results demonstrate how predictable, seemingly small shifts in an LCE fabric geometry, can impact the actuated textile’s blocking force.

### Integration of resistive heating elements for controlled morphing

**Fig. 16:**
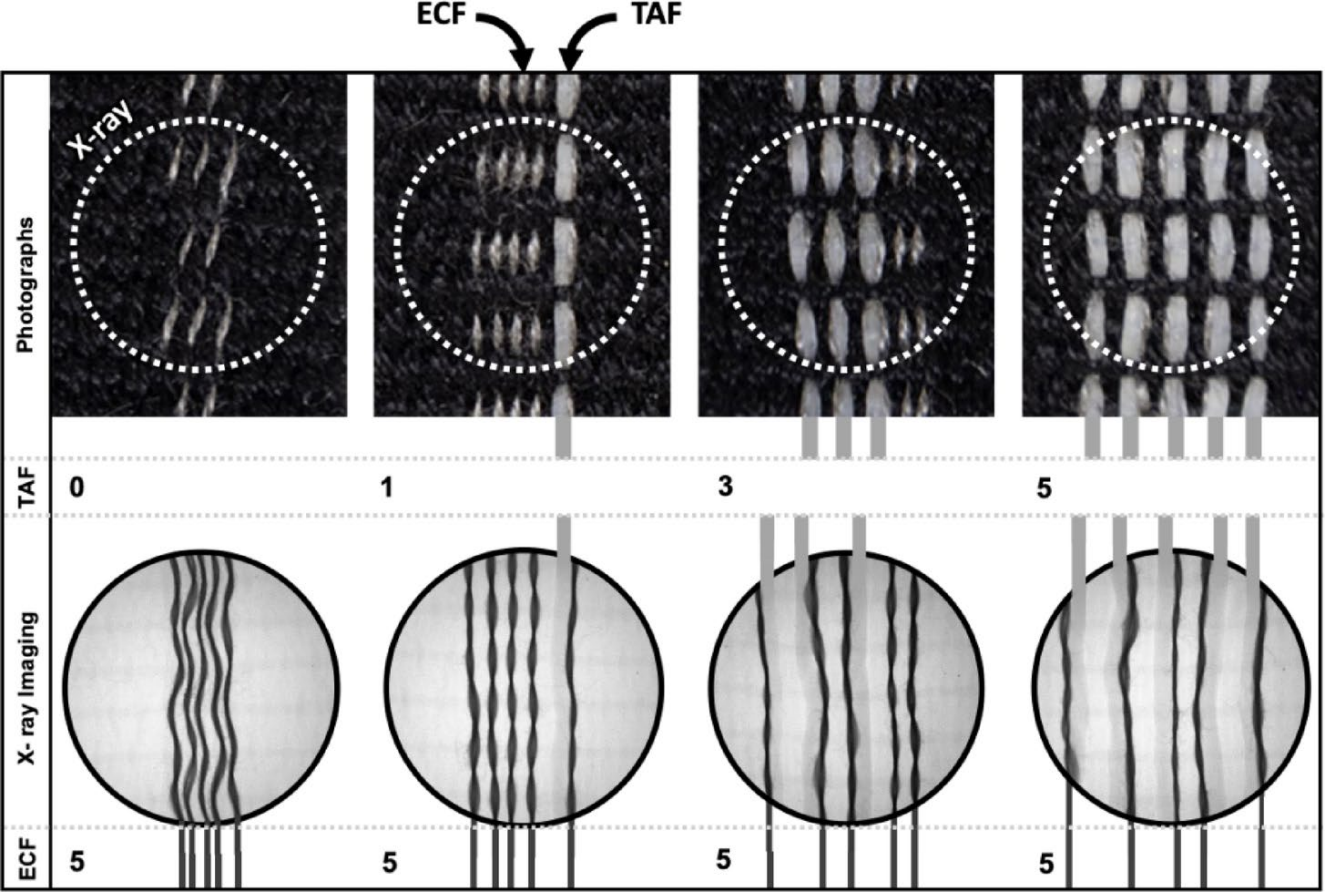
Photographs and their corresponding X-ray transmission images of four samples with zero, one, three, and five thermally actuating fibers (TAF), and five electrically conductive fibers (ECF). In these images, the circular regions each measure ca. 10 mm in diameter.

To demonstrate this design freedom and for the production of more complex on-demand self-morphing textile constructs, we next incorporated conductive fibers into our textiles which functionally behave as internal resistive heaters. When electrically conductive fibers (stainless steel thread) and thermally actuating fibers (LCE) are woven together, the different functional fibers can be difficult to visualize due to weft density and weave structure. To address these limitations, X-ray imaging was used to first characterize the spatial position of different fiber compositions within a series of plain woven textile samples (Fig. 16). For these different samples, the number of electrically conductive fibers (five) was consistent across the four samples, while the number of LCE fibers varied from zero to five.

**Fig. 17:**
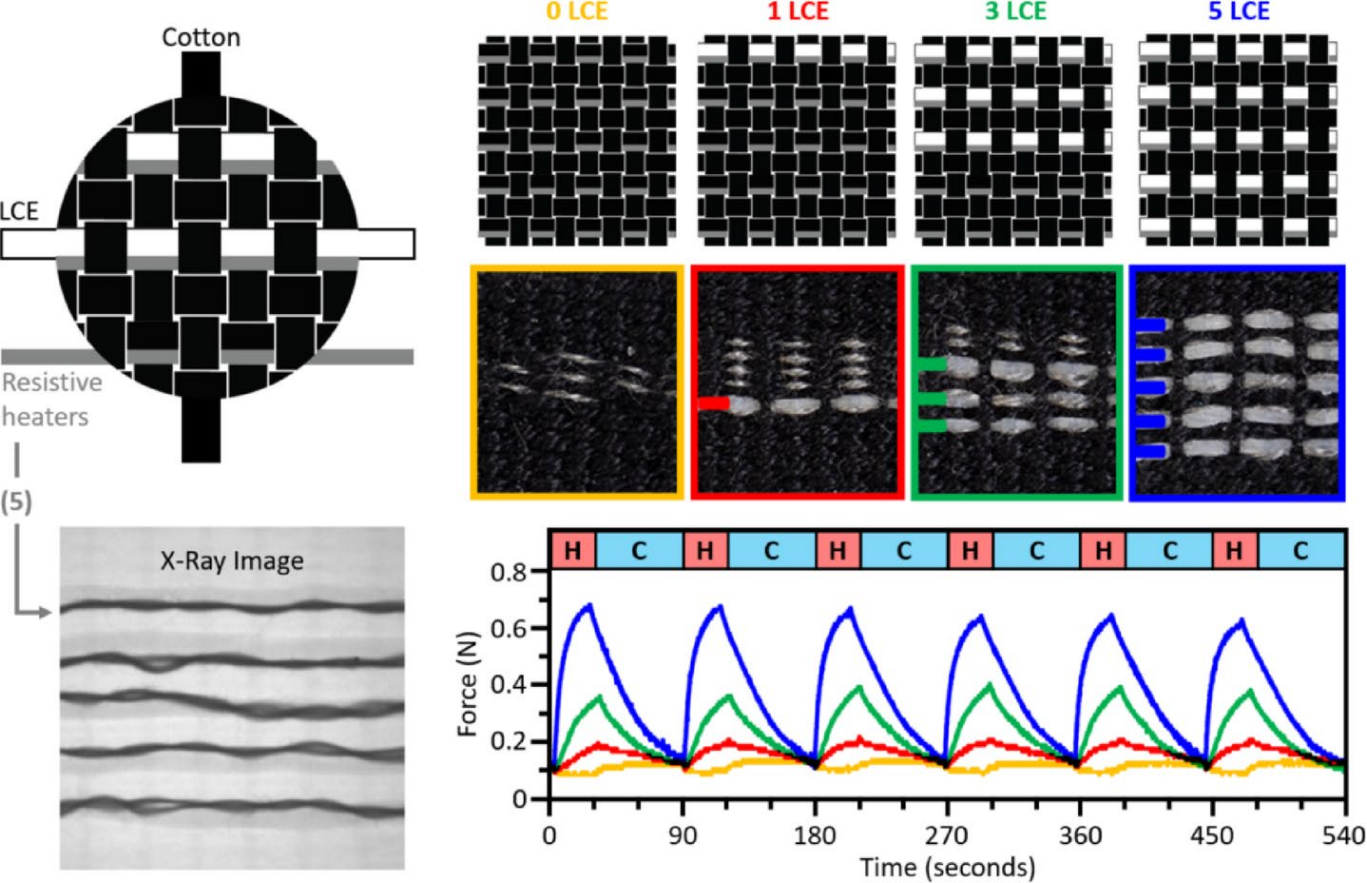
Overview of blocking force characterization for four plain weave samples with five integrated rows of conductive thread (resistive heaters), and 0, 1, 3, and 5 rows of LCE fiber. Left to Right: Grayscale legend identifying the different woven components and a corresponding x-ray transmission image showing the five conductive threads in detail, schematics and photos of the fabricated samples, and their corresponding blocking force measurements over 6 consecutive 30 s heating (H) and 60 s cooling (C) cycles.

To quantify how this increase in LCE fiber number affected exerted forces and actuation speeds, we performed a series of actuation force measurements on different fabric samples (Fig. 17). Sets of four plain woven samples (see Fig. 16) were fabricated, each of which measured 8.5 cm x 1.5 cm in size, such that direct comparisons could be made. Each sample contained five weft rows of electrically conductive stainless-steel fibers staggered with increasing amounts of LCE fiber per weft row (Fig. 17). One sample contained no LCE fibers, and five rows of conductive fiber, and the other three samples contained 1, 3, and 5 rows of LCE fibers, with the same five conductive wefts. The samples were repeatedly power cycled (0.5 A at ~ 12 V for 30 s, 0 A for 60s), and the average measured force for the 1, 3, and 5 fiber samples were 0.116 N, 0.337 N, and 0.655 N, respectively. Despite being hand-woven samples, these results demonstrate a generally linear increase in generated force with increasing numbers of actuating fibers. For the sample that contained no LCE fibers, a negative ‘pushing’ force can be observed during heating. Although cotton is described as an inert fiber, the sample containing no LCE fibers displayed a pushing force (or negative contraction) which can be attributed to the known expansion of the steel and cotton fibers^62^ when heated.

### Textile assemblies for application development

**Fig. 18:**
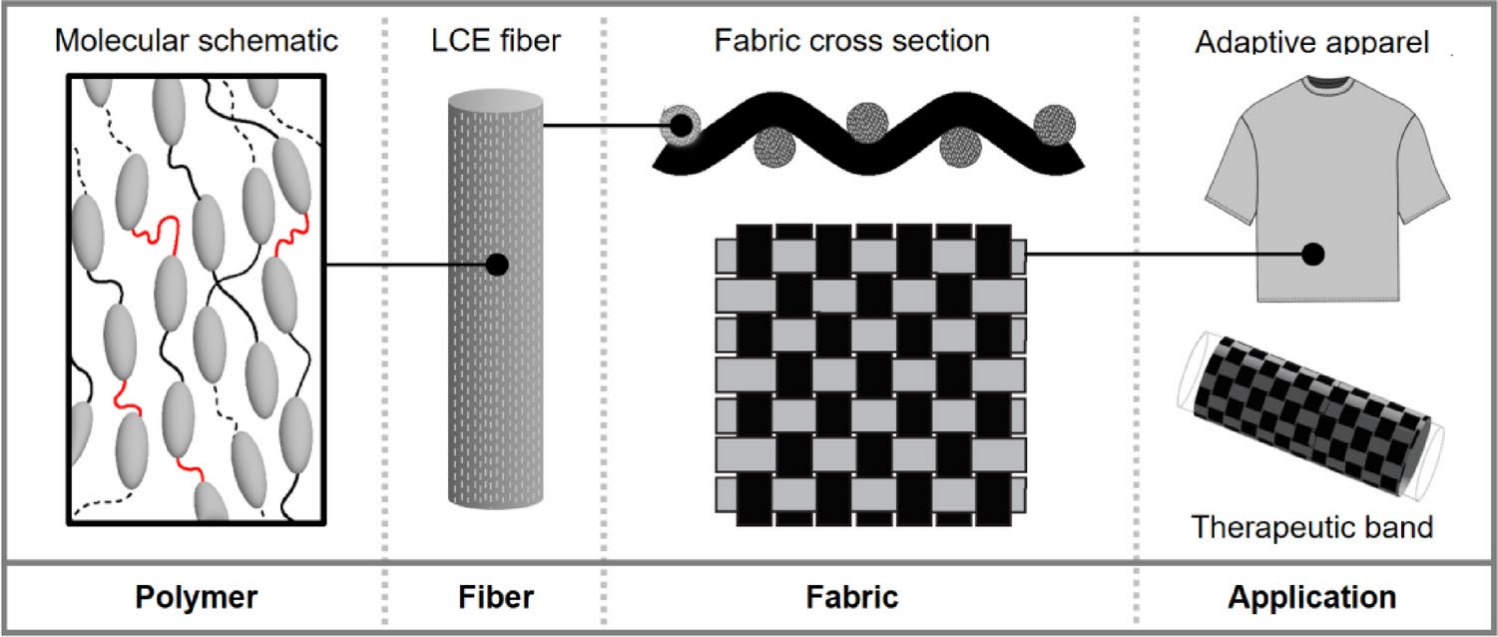
Structural hierarchy in LCE-based materials and devices.

Design decisions across multiple levels of the textile structural hierarchy (at the polymer, fiber, and fabric scales) can profoundly impact the performance of LCE-based textiles and devices (Fig. 18). On the polymer level, refinements to chemistry and chain length can impact mesogenic connectivity, phase transition temperatures, and chain alignment, which can in turn influence LCE performance and responsiveness to stimuli^60,61^. The subsequent integration of these active fiber elements with their passive (non-actuating) counterparts can be further leveraged to create material systems with pre-programmed behaviors for a potentially broad range of applications.

**Fig. 19:**
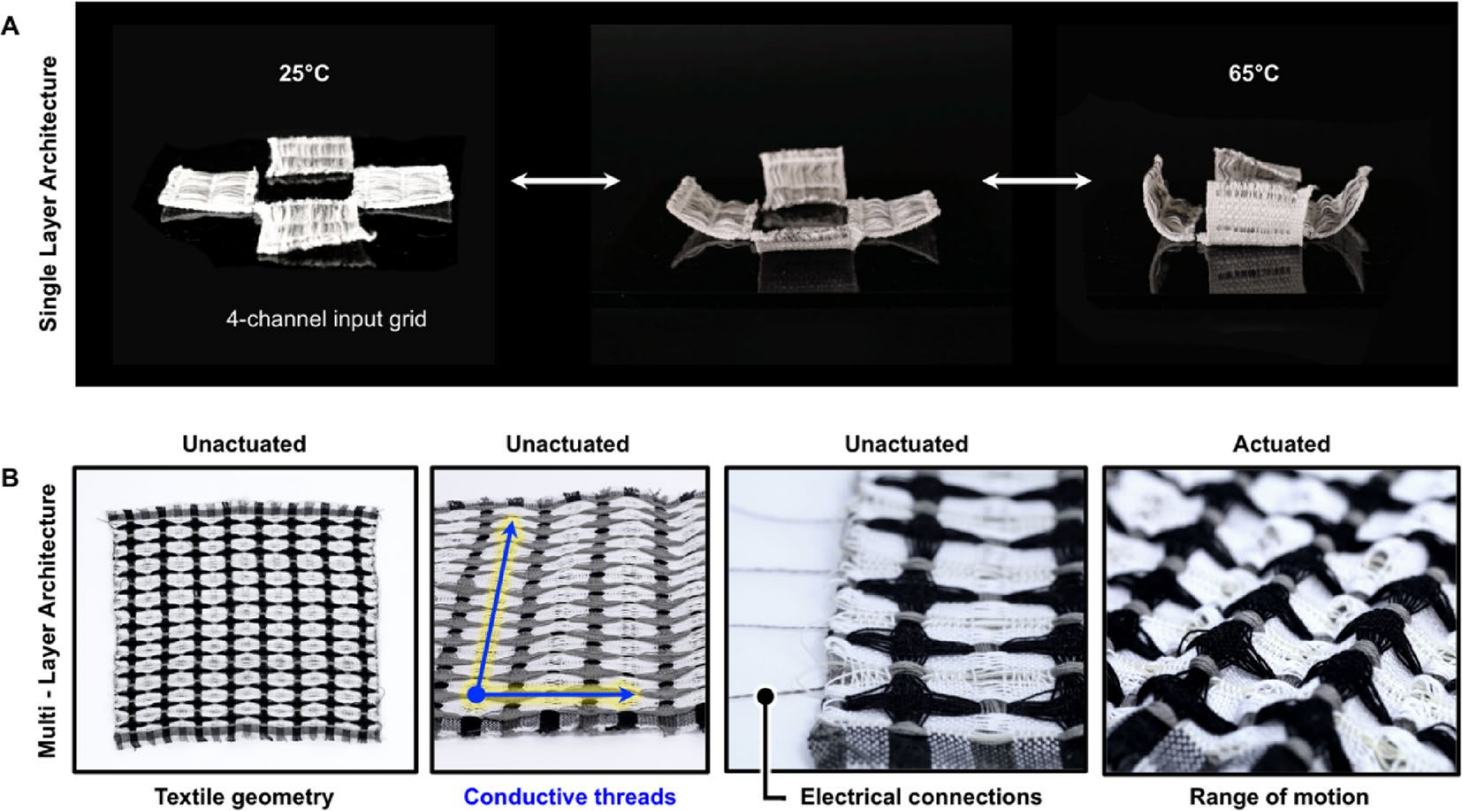
Assembly of textile elements for the generation of more complex devices across single-layered architectures organized into a self-folding box-like form (**A**), and multi-layered architectures organized into a programmable grid-like array of individually addressable elements (**B**). For A, each square face of the box measures ca. 4 × 4 cm, and for B, the diameter of each circular feature is ca. 3 cm, and when actuated, exhibits a vertical displacement of ca. 2 cm.

To explore the diverse application space for different actuatable textile architectures, we next created three functional prototypes, which exhibited different levels of actuation and programmable control. In the first example, Swedish lace samples were fabricated with LCE fiber, conductive threads, and cotton thread to create panels that could be individually actuated through a 4-channel electrical controller (Fig. 19A). Leveraging the ability of single-layer fabrics to curl upward and reverse back to a flat plane, panels were gradually actuated between temperatures of 25 °C and 65 °C (as measured with a thermal camera). The actuation of thermo-responsive active fibers occurs across a range of temperatures, where actuation begins at a lower temperature and ends at a higher temperature. This temperature range varies depending on the material employed. For example, SMAs, due to a narrow temperature range, generally exhibit a binary ‘on and off’ actuation^50^. In contrast, the LCE composition we employed in the present work operates across a ca. 35 °C temperature range^43^. While this large temperature range means that we cannot achieve the swift actuation possible with SMAs, as demonstrated here, partial heating affords a range of actuation displacements.

While Fig. 19A displays heating elements only embedded along the weft of the fabric, conductive elements can also be integrated along both the length and width of the woven fabric (Fig. 19B). When paired with LCE fibers, this design creates a transforming heat-responsive matrix, where actuation can be controlled based on the placement of overlapping conductive warp and weft threads^63–66^. The interlacing of warp and weft yarns at 90° angles allows for a conductive patterning that can be mapped and controlled digitally. Figure 19B demonstrates a double-deflected weft fabric with location-specific puffing and curling, providing a tractable experiential framework for the future development of fabrics with actuating and sensing matrices. This ability to regio-selectively control the actuation of a woven form may allow for the development of multi-layered, reconfigurable fabric shape displays that actuate over time based on designed inputs^29,64,65^, or for potential for use in responsive garments, such as wearables that use selective warmth for therapeutic applications.

**Fig. 20:**
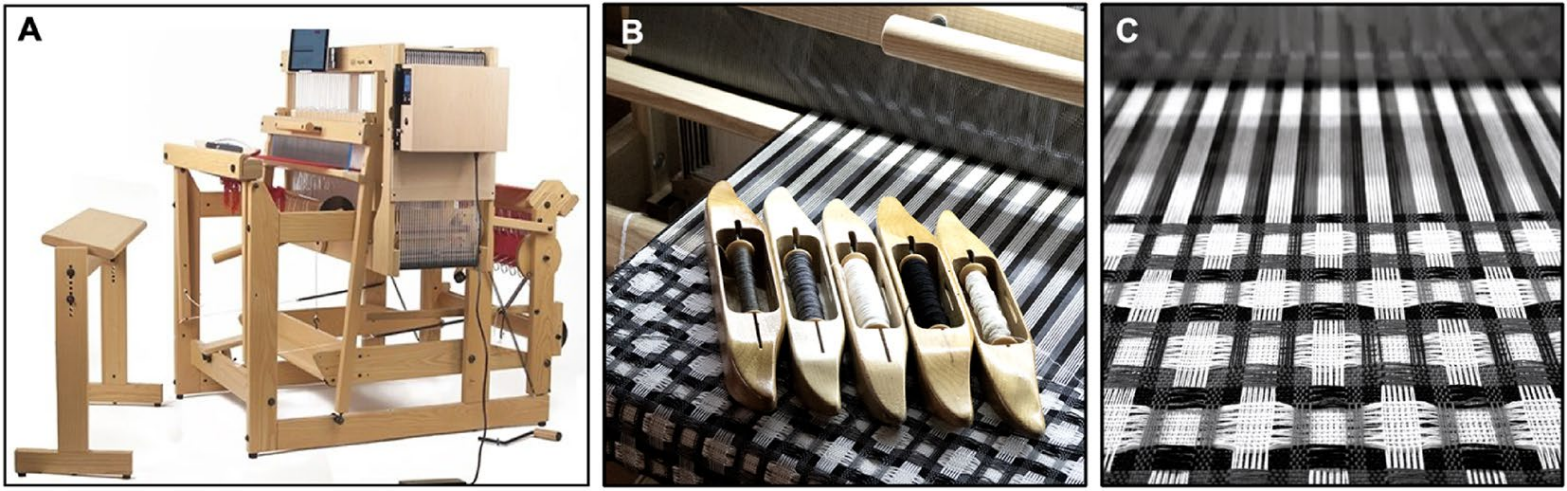
Overview of the process for producing 5 yards of LCE-containing fabric on a digital loom. (**A**) photograph of the dobby loom used to create the fabric, (**B**) detail of the 5 different fiber types employed in the design, (from left to right) conductive thread, gray cotton, white cotton, black cotton, and LCE fiber, (**C**) magnified view of the double woven fabric on the loom.

For our third functional prototype, we utilized a 16-harness dobby loom (Fig. 20A) to fabricate 5 m of fabric (Fig. 20B, C) for the production of a fully fashioned garment. Unlike a jacquard loom, a dobby loom allows the weaver to manually adjust the tension and placement of weft threads through physical intervention. The digital automation of the warp and physical control of the weft is ideal for rapid prototyping with novel weft fibers.

Primarily used for industrial production, digital looms utilize a bitmap-based user interface (Figs. 6 and 7, S6, S7) and an automated actuation system to control the position of individual warp (longitudinal) and weft (lateral) threads. The direct translation of digital information into physical form allows for faster fabrication through the automation of the handweaving process (Figures S8–S18)^5,6,26,67^.

**Fig. 21:**
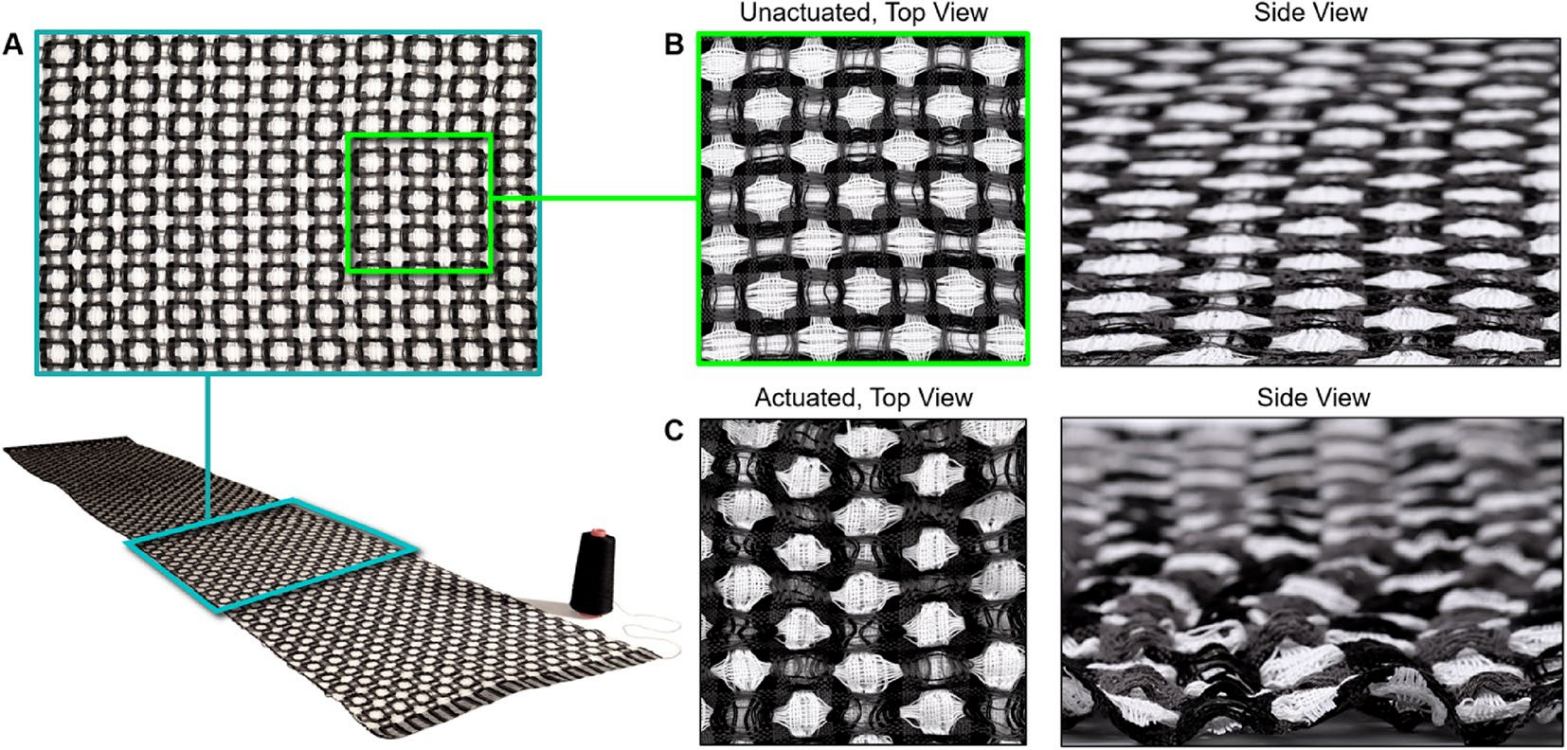
Overview of actuation behavior of a large-scale LCE-containing fabric. (**A**) Magnified plan view (upper) of a 1 m wide by 4 m long LCE fabric (lower), with a spool of thread for scale. Plan and lateral views of the unactuated (**B**) and actuated (**C**) LCE fabric, showing the resulting deformation in pattern and topology.

**Fig. 22:**
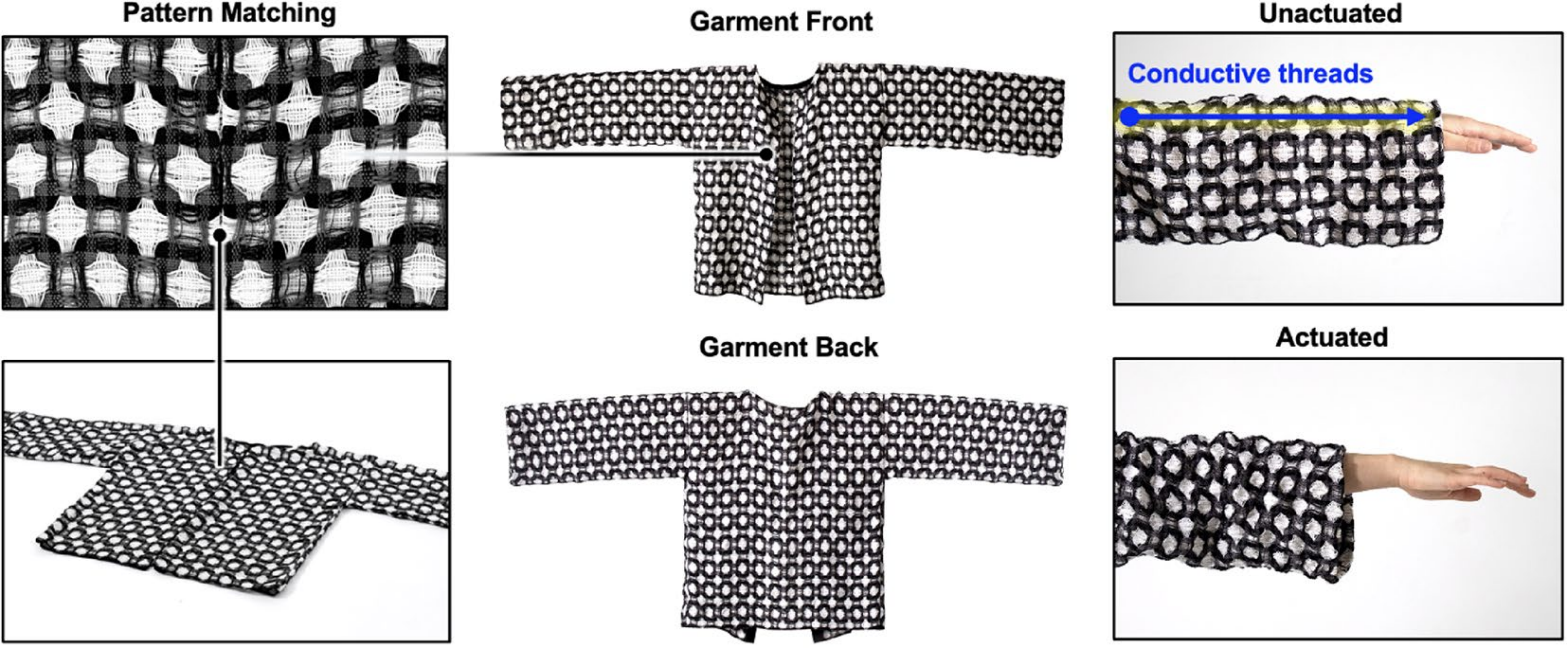
Overview of our functional garment prototype, detailing pattern matching for precise actuation, the front and back of the garment, and the internal thermally induced actuation (via resistive heating) of one sleeve of the garment.

During the production of our garment, we drew design inspiration from a critical historical reference. *The Shuttle Craft Book of American Handweaving* by Mary Meigs Atwater^62^. Example weave drafts Atwater’s book can be found in Figure S19. The inspiration for the design in Figs. 21 and 22 was a drawing of a weave draft from 1928. Beyond its connection to historical work, this double-deflected weave structure was chosen due to its programmable puffing and shrinking behavior (Fig. 18).

Programmable fabrics present an opportunity to develop wearables with novel functionalities, including active tailoring to specific stimuli. Figure 22 implements the final stage of the hierarchical schematic outlined in Fig. 18, by developing a wearable application. This application demonstrates the compatibility of LCE fabrics with industrial sewing machinery; an industrial sewing machine and serger were used to construct the fully fashioned jacket shown in Fig. 22. Pattern matching, or aligning specific visual components of the fabric, was a critical consideration for ensuring uniform actuation across all seams. In line with the observations reported in Figs. 11 and 21, shape-changing behaviors such as puffing and shrinking could be controlled across the garment through electrical implementation. By integrating the design and fabrication lessons learned from this study, we were able to demonstrate how electrically controlled (heated), woven LCE textiles may be used in active tailoring, thus laying the groundwork for application development in fields ranging from site-specific therapeutic heating to shape-changing garments.

## Discussion and future work

Our present study expands on the prior design space for single-layered LCE fabrics by investigating the properties of new woven LCE textile geometries, demonstrating how predictable, subtle shifts in fabric structure impact an actuated textile’s geometric transformations and block force generation. Single-layered fabrics can exhibit in-plane shrinkage and rotation when actuated, while multi-layered fabrics can curl and expand when actuated. These types of transformational behaviors can be used singly or further combined for the generation of more complex forms and devices that exhibit modularity and matrix-enabled actuation, having relevance for applications in therapeutics, deployable structures, thermoregulation, and soft robotics. Responding to the need for tools that support the development of actuating textiles programmed at the material level, this work further contributes to the vision of intelligent, stimuli-responsive fabrics by describing how deformation forces vary across a wide range of woven structures.

Variables that could be considered in future work include different loom types, fiber types, fiber coatings, warp selections, weft deliveries, and fabric finishings. On the fiber level, variations in parameters related to fiber twist (twists per inch) and extrusion methods (nozzle shape, speed, and material cross-section) could be employed to tune the behavior of the resulting fabric^20,36^. Recent advances in LCE chemistry also point toward the possibility of synthesizing these fibers with more sustainable chemistries that can biodegrade, which aligns with current trends and concerns in the textile industry^4^. These improvements may also support the development of fibers that are compatible with the tension requirements of specific textile industrial machinery, such as a jacquard loom, which would directly impact the translational nature of this research, enabling the scaling of technology from the lab to industry. A broader exploration of weave structure across the textile design space, including building on previous work in embroidery and knitting, could lead to a more holistic understanding of how LCE fibers can enable shape-changing behavior. In addition, the incorporation of many different environmentally responsive fibers into a single textile could be employed to create multiple actuation modes that respond to specific environmental stimuli. The aforementioned avenues for future development are fundamentally tied to the long history of fabric construction, highlighting how the ubiquity of textiles provides a one-of-a-kind opportunity to develop novel technologies that feel familiar.

## Materials and methods

### Illustrations

All of the 2D schematics shown in the figures were created using Adobe Illustrator (Adobe Systems), and all of the 3D renderings were created using Rhinoceros 8 (Robert McNeel & Associates).

### Fiber chemistry

Following the formulations introduced by Forman et al.^43^, the following raw materials were used for the creation of heat-responsive LCE fibers: RM82 (1,4-Bis-[4-(6-acryloyloxyhexyloxy)benzoyloxy]-2-methylbenzene), RM257(Bis-[4-(3-acryloyloxypropyloxy)benzoyloxy]-2-methylbenzene), EDDET(2,2-(ethylenedioxy)diethanethiol); GDMP(glycol di(3-mercaptopropionate)); ATATATO(1,3,5-triallyl-1,3,5-triazine-2,4,6(1 H,3 H,5 H)-trione), DPA (Dipropylamine), I-651 (2,2-dimethoxy-2-phenylacetophenone), and BHT (Butylated hydroxytoluene). EDDET, TATATO, BHT, I-651, and DPA were purchased from Sigma Aldrich. GDMP was purchased from Fisher Scientific. RM82 and RM257 were purchased in small quantities from Sigma Aldrich and in bulk from Daken Chemical^33^.

### Fiber production

The same method from Forman et al.^43^ was used for fiber produciton. 15 g of RM82 and 0.105 g of BHT were added to a glass vial and placed in a toaster oven until melted^43^. 4.5 mL of EDDET and 800 µL of TATATO were added to the vial and mixed. The vial was returned to the oven and heated until the mixture was completely clear. 0.42 g of I-651 and 100 µL of DPA were then added to the vial and vigorously mixed. The resulting solution was then centrifuged at 3,000 RPM for 3 min to remove any air bubbles^43^. The machine used to create the fibers is shown in Fig. 5A. This machine contained a temperature-controlled syringe pump for extruding high-viscosity fluids, a drawing spool to align and thin the fibers, and UV light channels to crosslink and cure the fiber. Fibers were dip-coated in mineral oil and then coated in talc powder. A UV-blocking enclosure was used to ensure safety.

### Loom details

A 24-inch 16-shaft Ashford table loom was used for the fabrication of samples described in this work. A handloom was chosen because it allows for a high level of control over fabric properties, flexibility in fabric design compared to industrial counterparts, and experimental opportunities for exploring active fibers and weaving techniques. Handlooms are particularly suitable for small-scale production and allow for close monitoring of quality control throughout the weaving process.

A 12-dent stainless steel reed was used for all samples. For single and double-layered designs, only 8 shafts were employed. The design process of this work involved exploratory experimentation of LCE integration into advanced weave structures and was informed by the authors’ formal education and professional experience in textile engineering, material science, and robotics.

A 16-harness electronic Louet dobby loom was utilized to create the large-scale samples and garment shown in Figs. 20 and 21, and 22. An electronic dobby loom is a type of loom that utilizes a dobby mechanism to create complex patterns through the automated control of individual warp threads; the term dobby is derived from “draw boy,” a weaver’s assistant who would manually lift the warp threads. An electronic dobby uses computerized technology to control the weaving process, and warp threads are controlled by a digital interface instead of manual operation. This design allows for greater precision when weaving and enables the ability to store woven patterns in specialized software. A dobby loom also allows for manual control of weft tension, which was critical for successfully weaving the LCE fibers presented in this work.

### Fiber compositions

Electrically conductive fibers (stainless steel thread), thermally actuating fibers (LCE) and cotton threads were used to create the woven fabrics presented in this study. A stainless steel conductive thread (Dev-11791, Sparkfun) was used as an electrical component to trigger heat-responsive actuation in the warp and the weft; this fiber is flexible, has sufficient thermal resistivity, does not oxidize, and is commercially available. 10/2 cotton was used for both the warp and weft because of its thermal and electrical insulating properties. Cotton also has high thermal durability, a key feature for embedding conductive elements into woven textiles. We utilized a thermally actuating fiber, LCE, which contracts approximately 40% of its length when heated at 65 °C; this shape change reverses when cooled, and actuation can be repeated over many cycles with minimal breakdown in mechanical performance (Fig. 12).

### Electrical implementation

A Xiao ESP32S3 was used to digitally control fabric actuation. For multiple channels of actuation, a PCA9685 16-Channel 8 W FET driver with an IoT interface (National Control Devices) was controlled from a single power supply.

### Manufactured textile patterns

Textile structures were chosen for this work based on major categories commonly used in textile design, engineering, and prior work. Naming conventions used in textile communities, such as huck lace, Swedish lace, and double-deflected weft were employed and are defined below.

Plain weave is the most common weave structure, where each weft passes over a warp thread in an alternating sequence. This design results in a uniform textile, with a balance of strength and durability as the fabric is resistant to distortion. A spaced plain weave, or gauze weave, has the same weft structure as a regular plain weave, but the warp threads are grouped together by skipping a desired number of spaces in the reed; this design leads to a fabric with programmable permeability. This increased openness allows for enhanced drape, visual distinctness, and increased surface texture while maintaining fabric stability. A traditional leno weave twists adjacent warp yarns around a series of wefts; this is usually done with beads that are introduced when threading. A huck lace weave mimics the characteristics of a traditional leno weave with a simpler approach to construction that still results in the crisscrossing of warp yarns. Compared to a plain-woven fabric, a mock leno design has improved drape while still maintaining openness and structural stability. A Swedish lace fabric is characterized by woven floats and tie-downs that result in weft patterning. Woven floats are strategically placed to create various patterns, where the back of the fabric is the inverse of the front. Waffle weave structures include warp and weft floats and tie-downs, resulting in a textured surface that resembles a honeycomb. These recessed areas of the structure create a unique surface texture and topology that is breathable, insulating, structurally stable, and lightweight.

Multi-layered fabrics have increased thickness, strength, durability, and insulation; their unique properties are suitable for the creation of barriers with different material attributes. A puffer fabric is created through gaps or infrequent tie-downs, while a stiff fabric is created through two connected patterns that do not pull apart due to frequent tie-downs (this structure has been used most frequently in the e-textile literature). Other double-layered fabrics include double cloths, which provide more interconnection between the two fabric layers. A double-deflected weave has two layers that frequently interchange, while a double-woven fabric with floats is characterized by two separate fabric layers with float patterns that are not tied down or connected. Multi-layered fabrics have increased thickness, strength, durability, and insulation; their unique properties are suitable for the creation of barriers with different material attributes.

### Textile characterization

#### Mechanical testing

A CellScale Univert S mechanical testing device with a 1 N load cell was used for the contractile force measurement tests.

#### Optical imaging

An EOS Canon R50 was used for photography of the different textile samples.

#### Scanning electron microscopy (SEM)

All textile samples were imaged (uncoated) in low vacuum (15 Pa) with an acceleration voltage of 20 keV using a Tescan Vega GMU variable pressure scanning electron microscope. Elemental mapping was performed using a Bruker dual-detector energy dispersive spectrometer system to minimize shadowing artifacts.

#### X-ray imaging

X-Ray transmission images of the fabric samples were acquired from a PXi (Pacific X-Ray Imaging) GenX-130 flatbed imager.

## Electronic supplementary material

Below is the link to the electronic supplementary material.

Supplementary Material 1

## Data Availability

The datasets generated in this study are included in this published article (in its accompanying supplementary information document), and any additional details are available from the corresponding author upon request.
