# Supplementary material for: A framework for handweaving robotic textiles with liquid crystal elastomer fibers
Source: Sci Rep. 2025 May 15;15:16883. doi: 10.1038/s41598-025-97835-1 (PMC12081926; doi:10.1038/s41598-025-97835-1)
Supplement: Supplementary file 1 — Supplementary Material 1 [file 41598_2025_97835_MOESM1_ESM.pdf]

Supplemental Information

A Framework for Handweaving Robotic Textiles with Liquid Crystal Elastomer Fibers

Sarah Nicita[1], James C. Weaver[2], Hisoshi Ishii[1], Jack Forman[1]
1 Tangible Media Group, MIT Media Lab, Cambridge, MA, USA
2 Harvard's Wyss Institute for Biologically Inspired Engineering, MIT Department of Civil and Environmental Engineering, Cambridge, MA, USA

Supplementary Figures 1-19

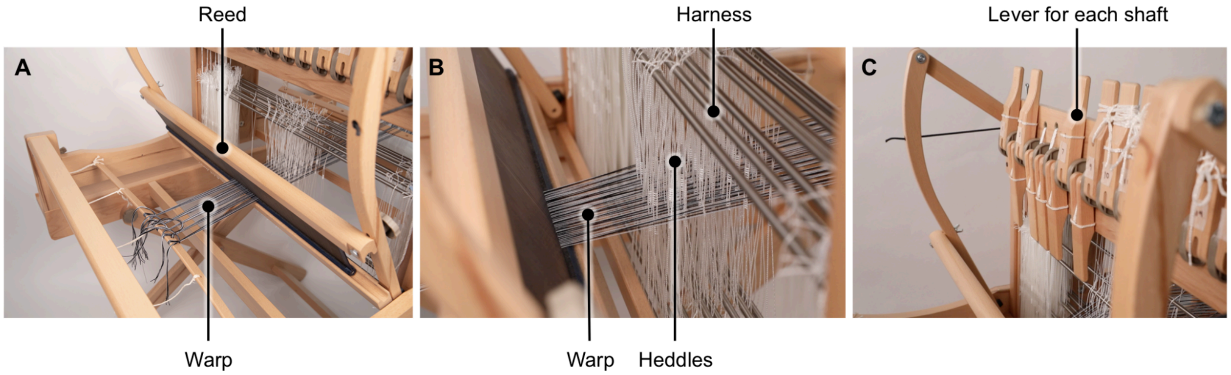

Supplementary Figure 1: Labeled components of a 16-harness table loom. A) the warp passing through the reed, B) the warp passing through heddles on 8 harnesses, C) the levers for each shaft, which control each harness.

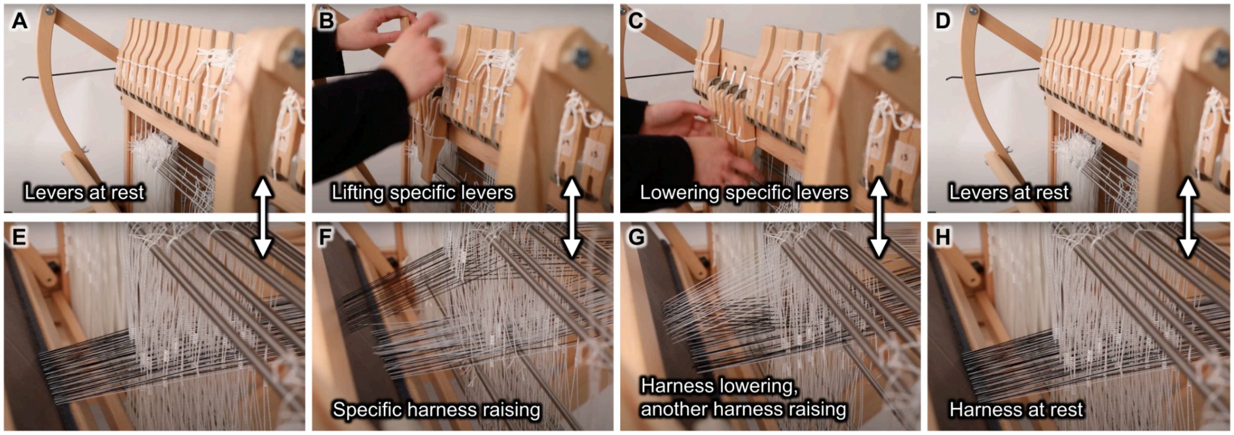

Supplementary Figure 2: Schematic illustrating the weaving process over time, with a focus on the relationship between moving levers and lifting specific warp ends. A) levers at rest, B) lifting specific levers to produce a specific woven pattern, C) changing these levers to create a second woven pattern, D) levers at rest.

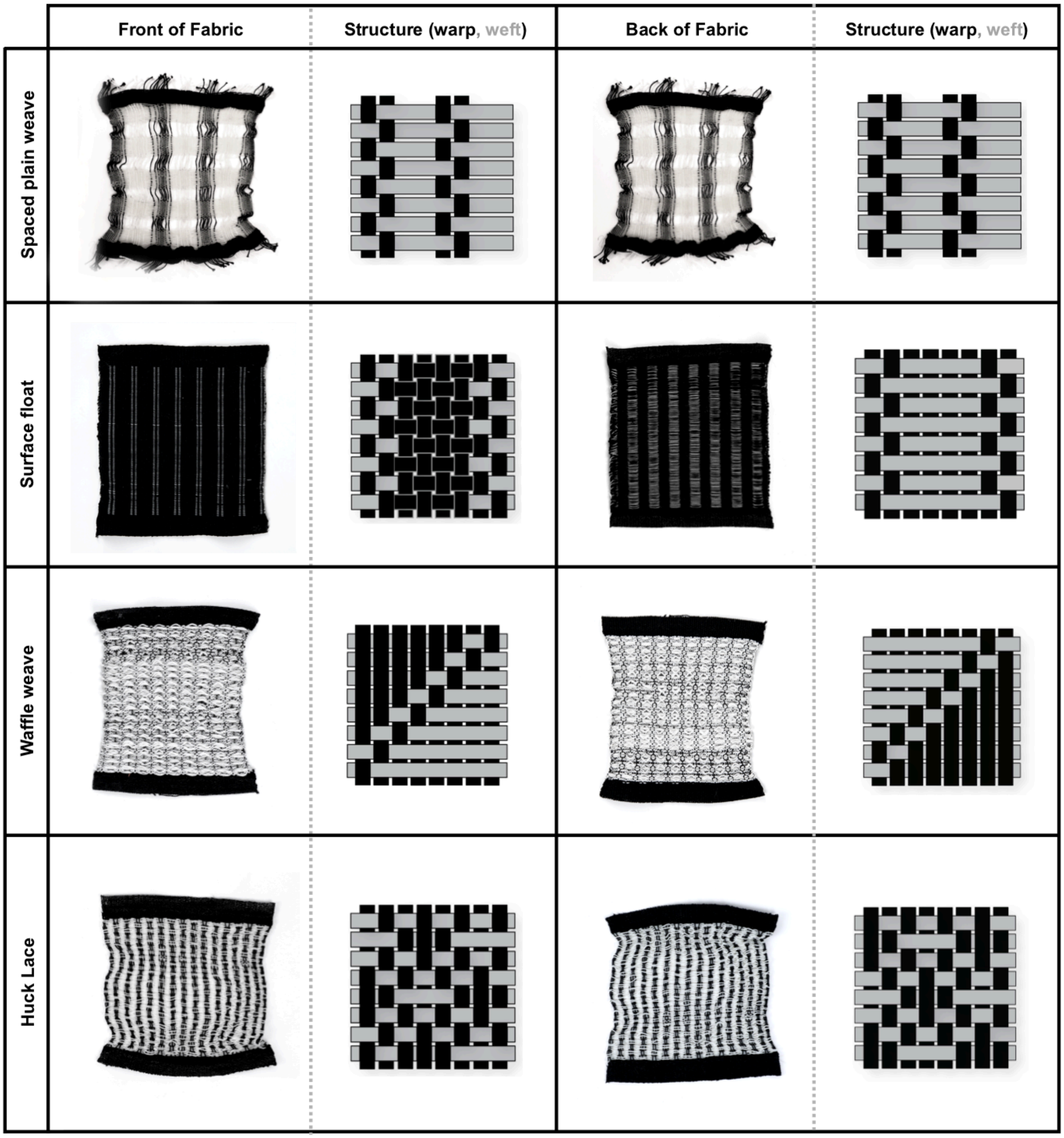

Supplementary Figure 3: Overview of the front and back yarn architecture of four single-layered fabrics, showing both the produced fabric and a corresponding magnified schematic of its woven structure.

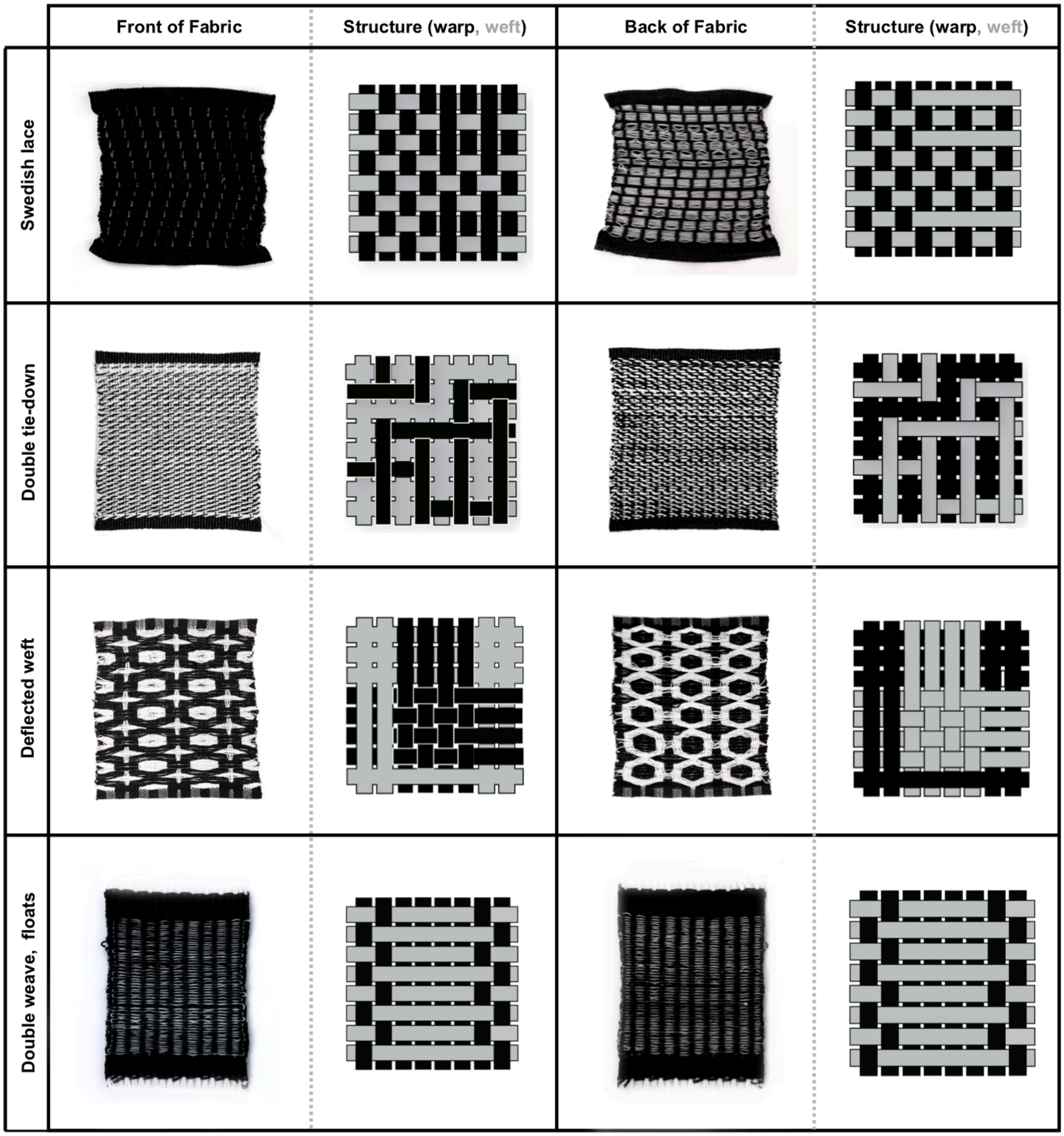

Supplementary Figure 4: Overview of the front and back yarn architecture for one single and double layered fabrics, showing both the produced fabric and a corresponding magnified schematic of its woven structure.

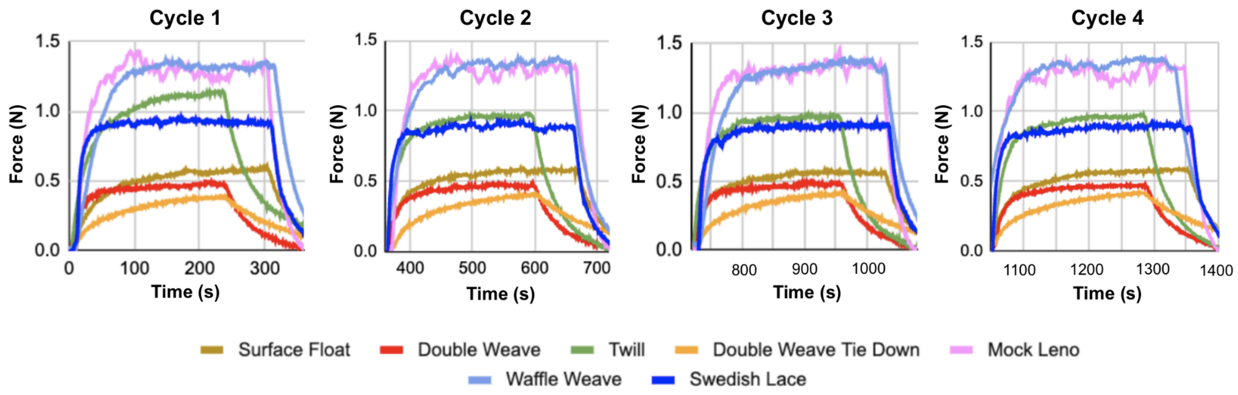

Supplementary Figure 5: Comparison of blocking force measurements across different textile geometries over four heating/cooling cycles.

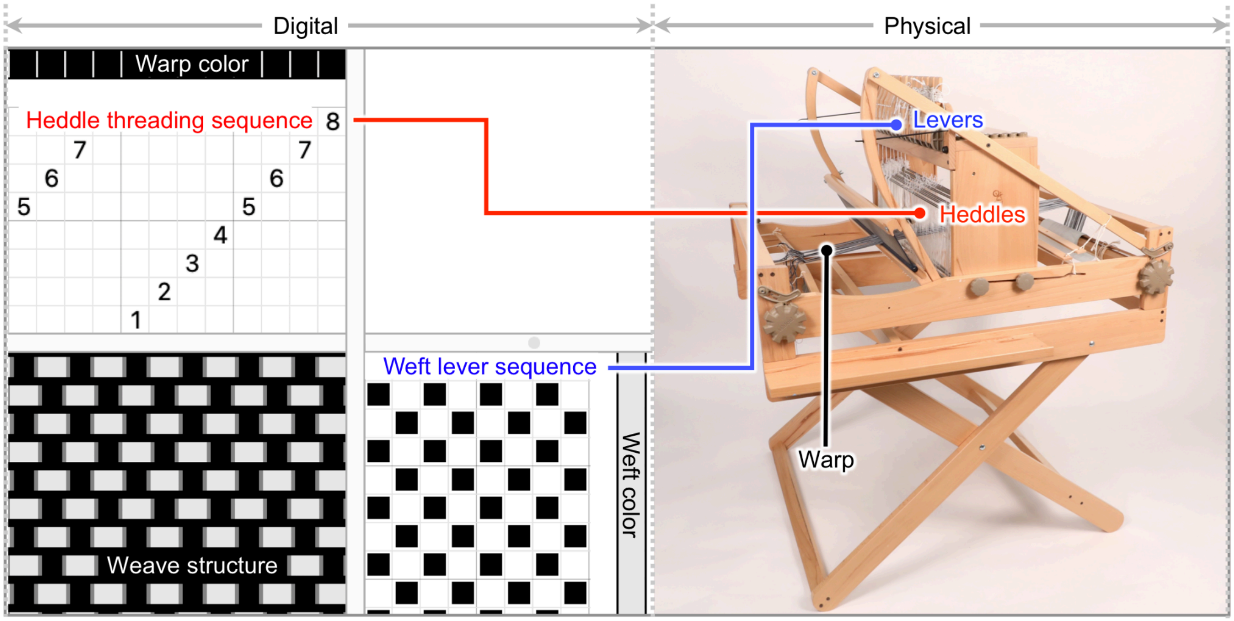

Supplementary Figure 6: Diagram associating components of a digital weave draft to those of a physical loom. The threading sequence is read from right to left and directly relates to the order in which the warp ends are brought through the heddles. The weft lever sequence is read from the top down, and indicates which levers should be lifted and lowered to create a specific weave structure. The weave structure is enabled by the combination of the of the heddle threading sequence and the weft lever sequence.

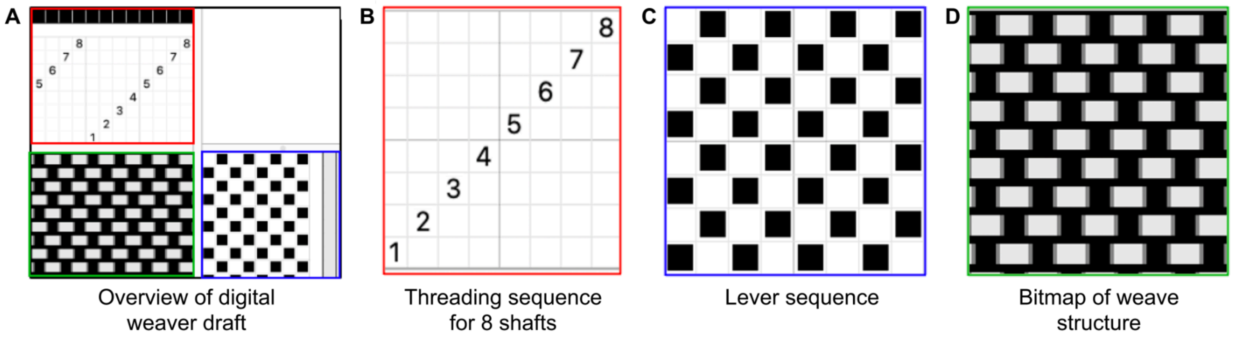

Supplementary Figure 7: Diagram identifying and naming components of a digital weave draft. A) overview of a digital weave draft, B) threading sequence for 8 shafts, C) lever sequence, D) bitmap of a weave structure.

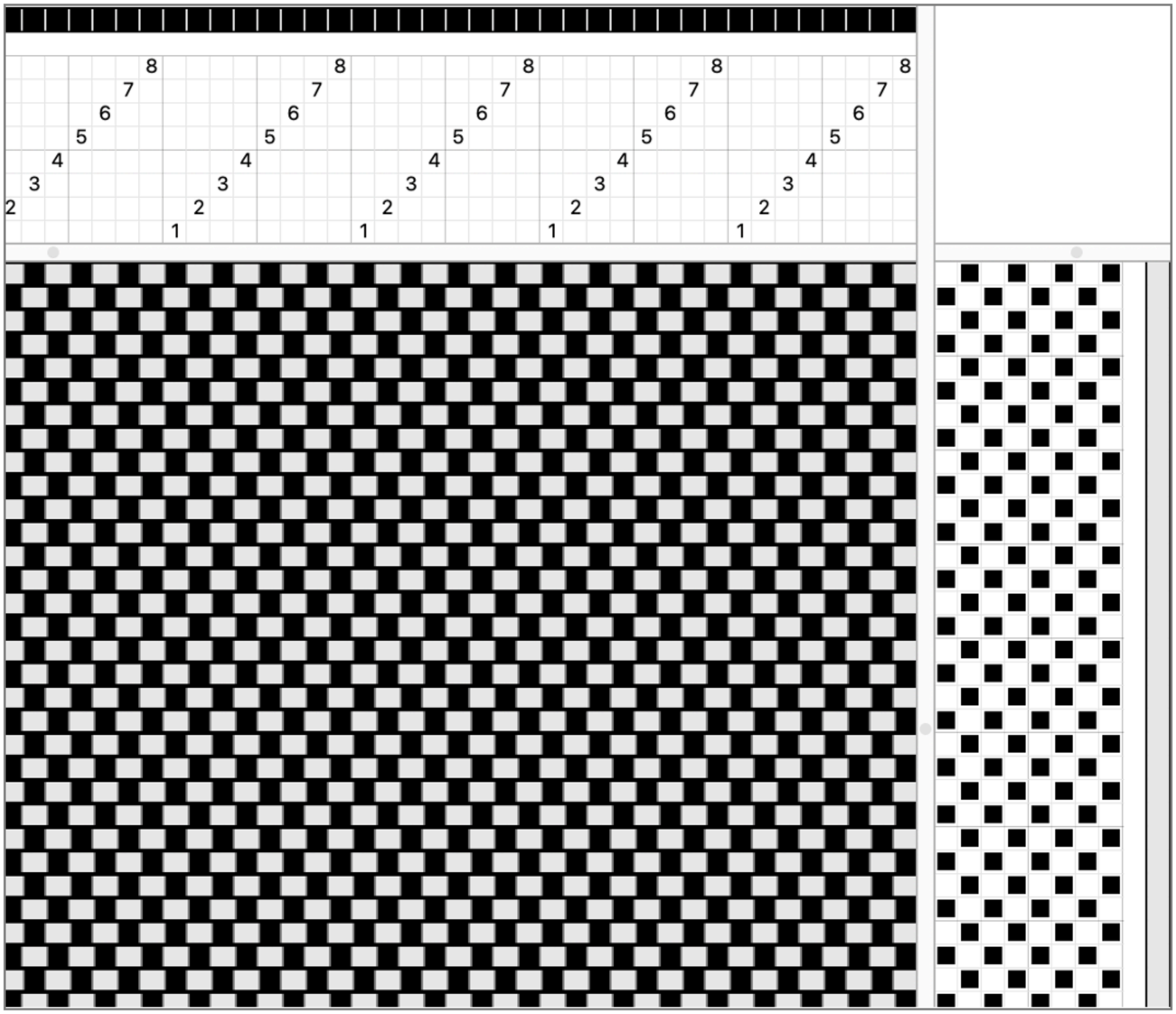

Supplementary Figure 8: Weave draft for a plain weave structure.

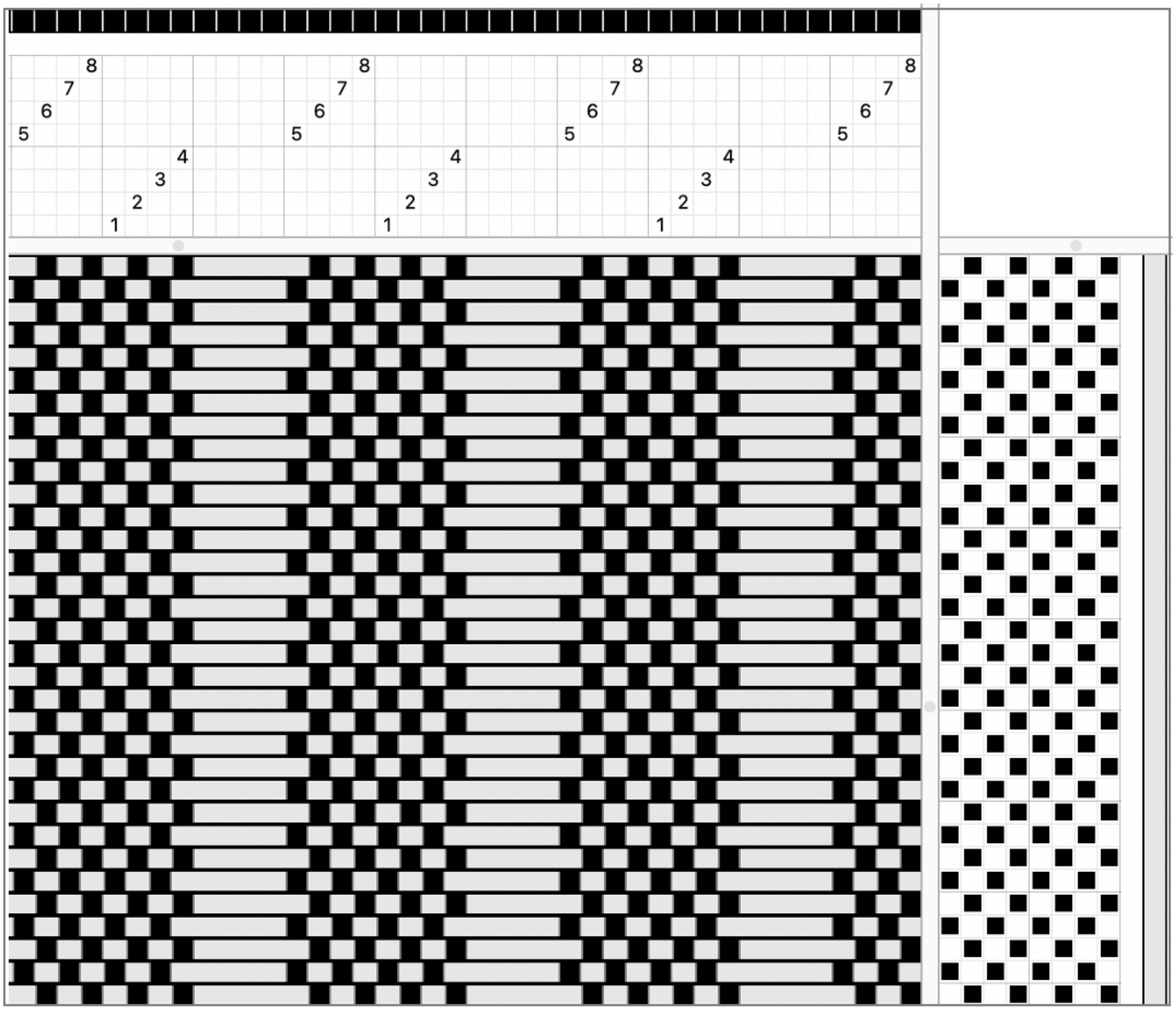

Supplementary Figure 9: Weave draft for a spaced plain weave structure.

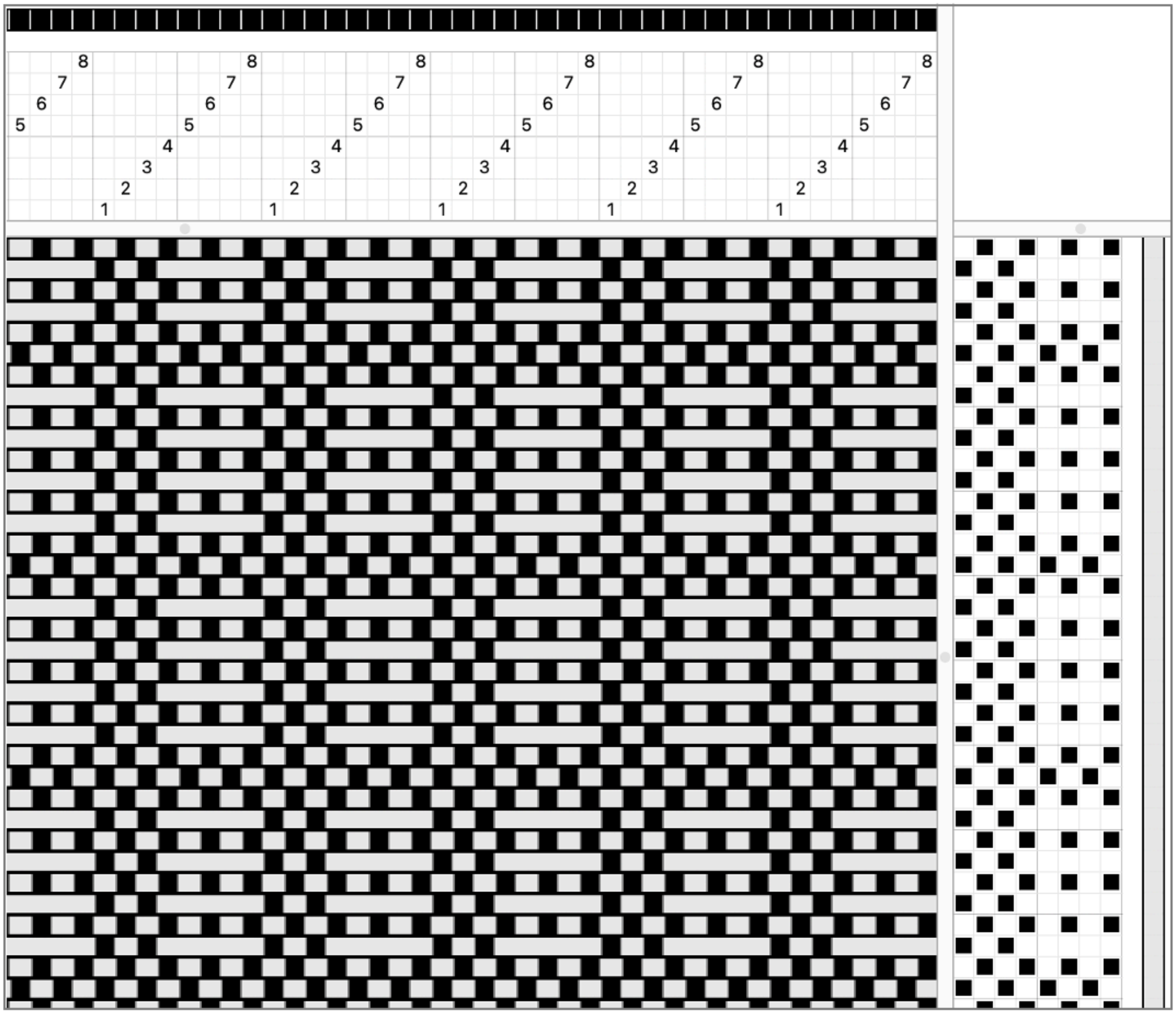

Supplementary Figure 10: Weave draft for surface float structure.

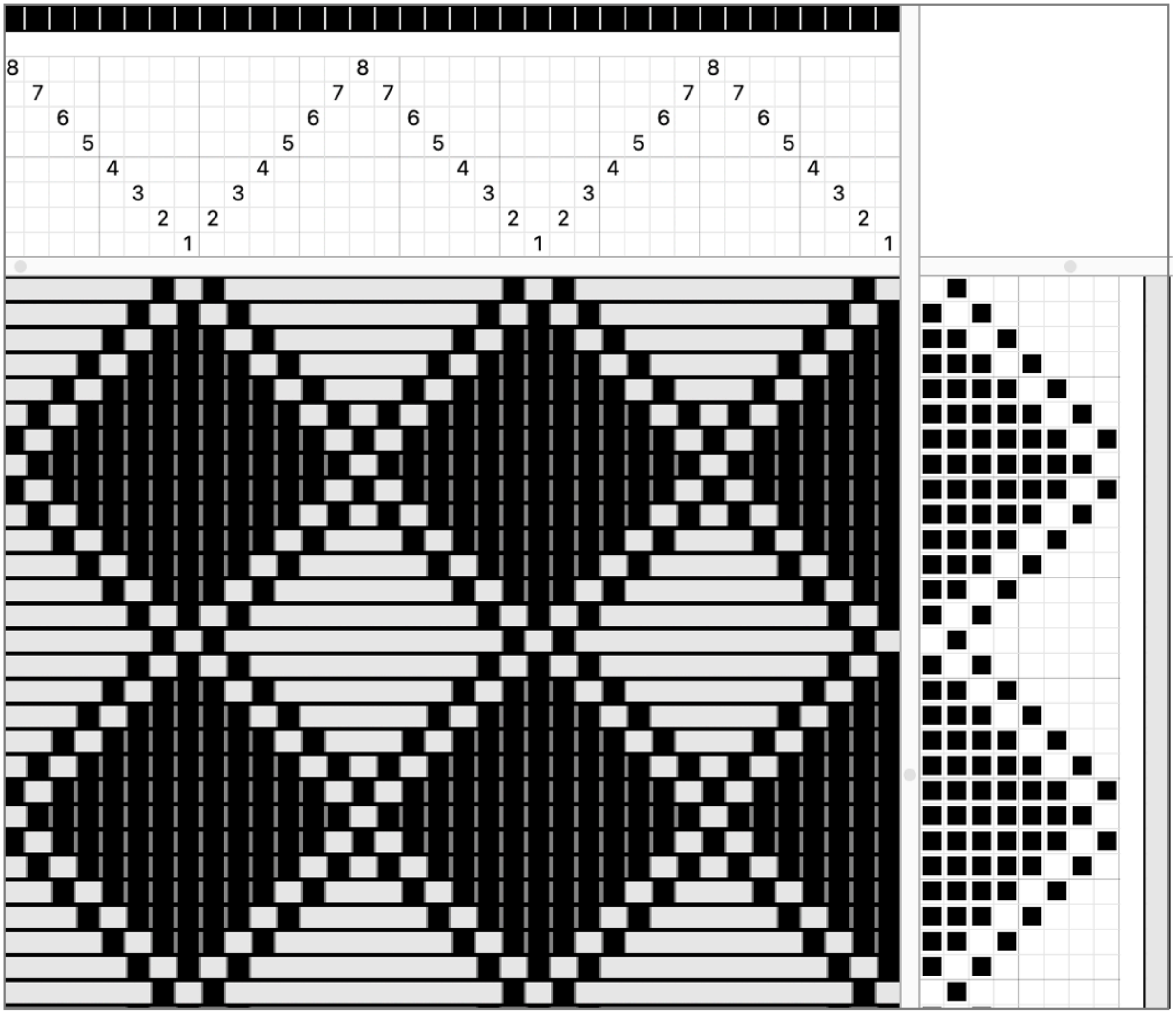

Supplementary Figure 11: Weave draft for a waffle weave structure.

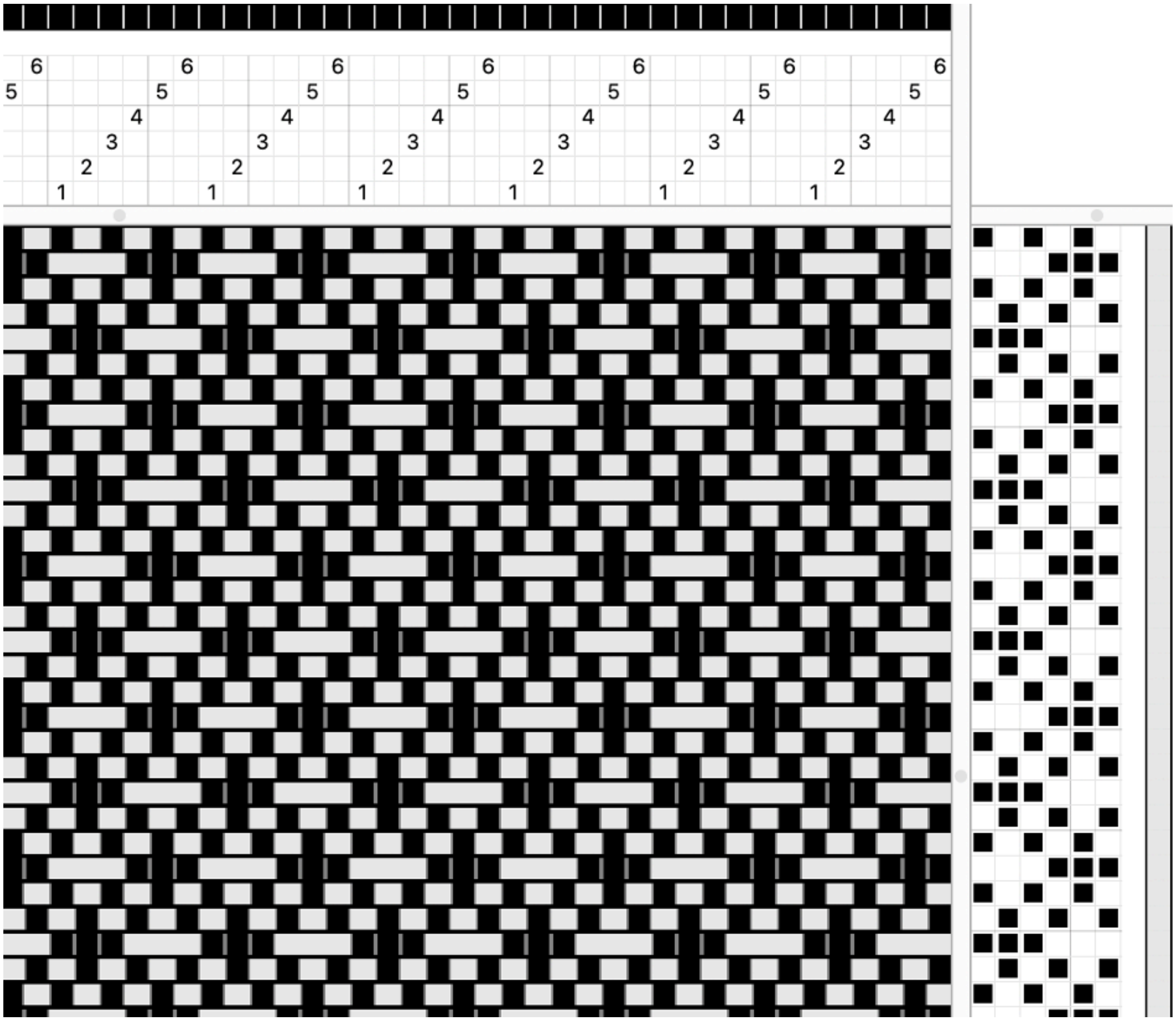

Supplementary Figure 12: Weave draft for a mock leno structure.

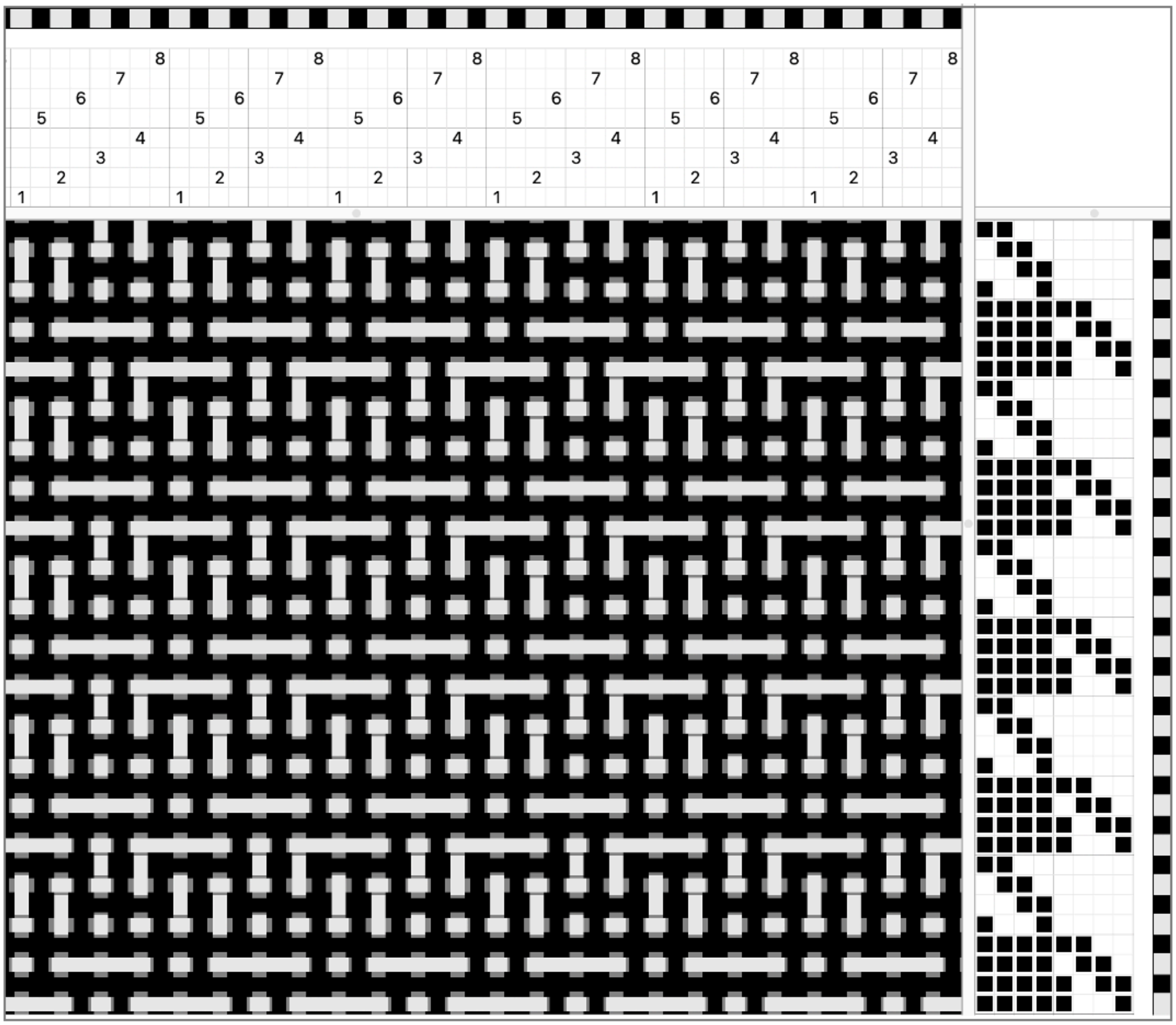

Supplementary Figure 13: Weave draft for one side of a double woven structure.

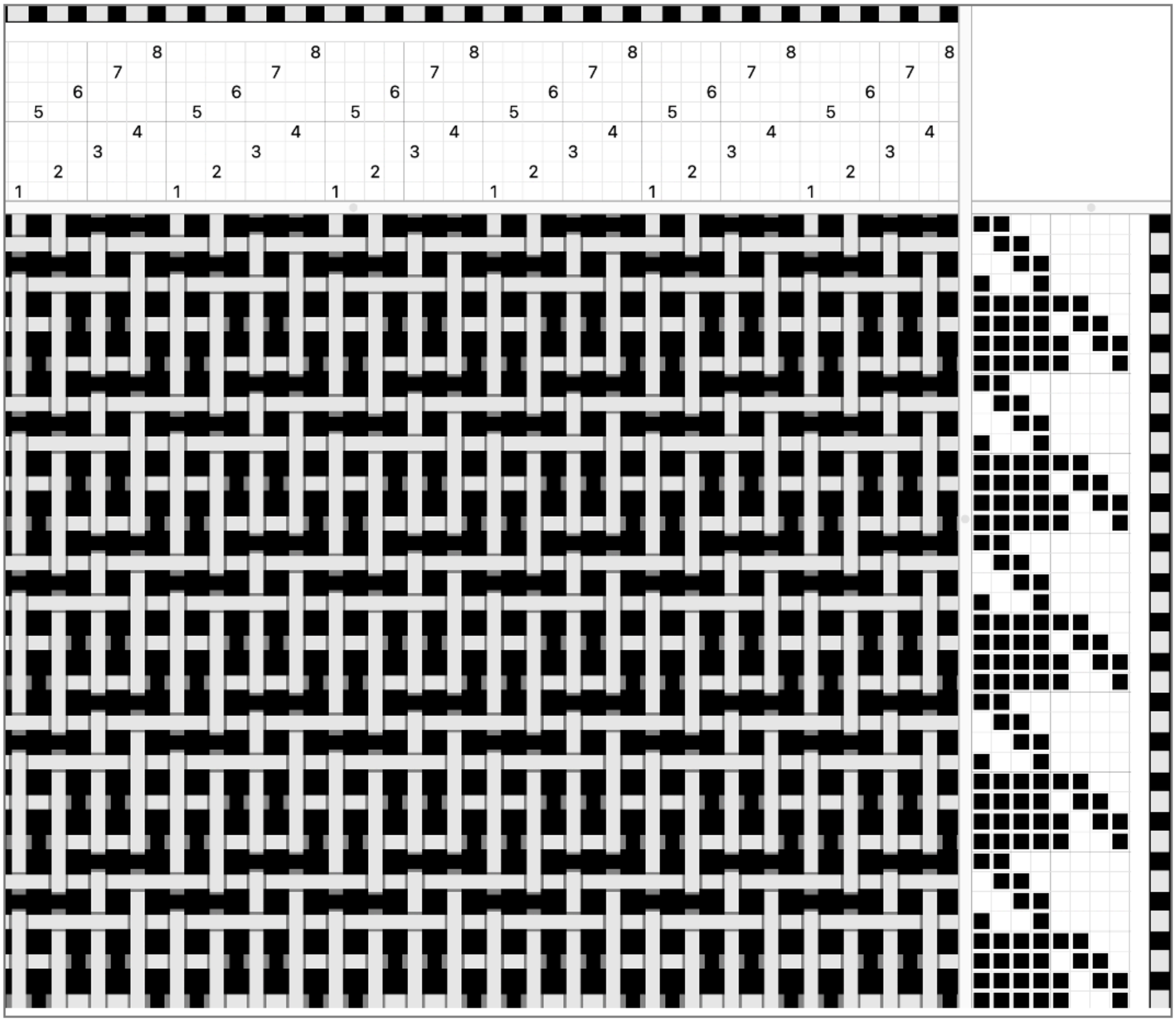

Supplementary Figure 14: Weave draft for the other side of a double woven structure.

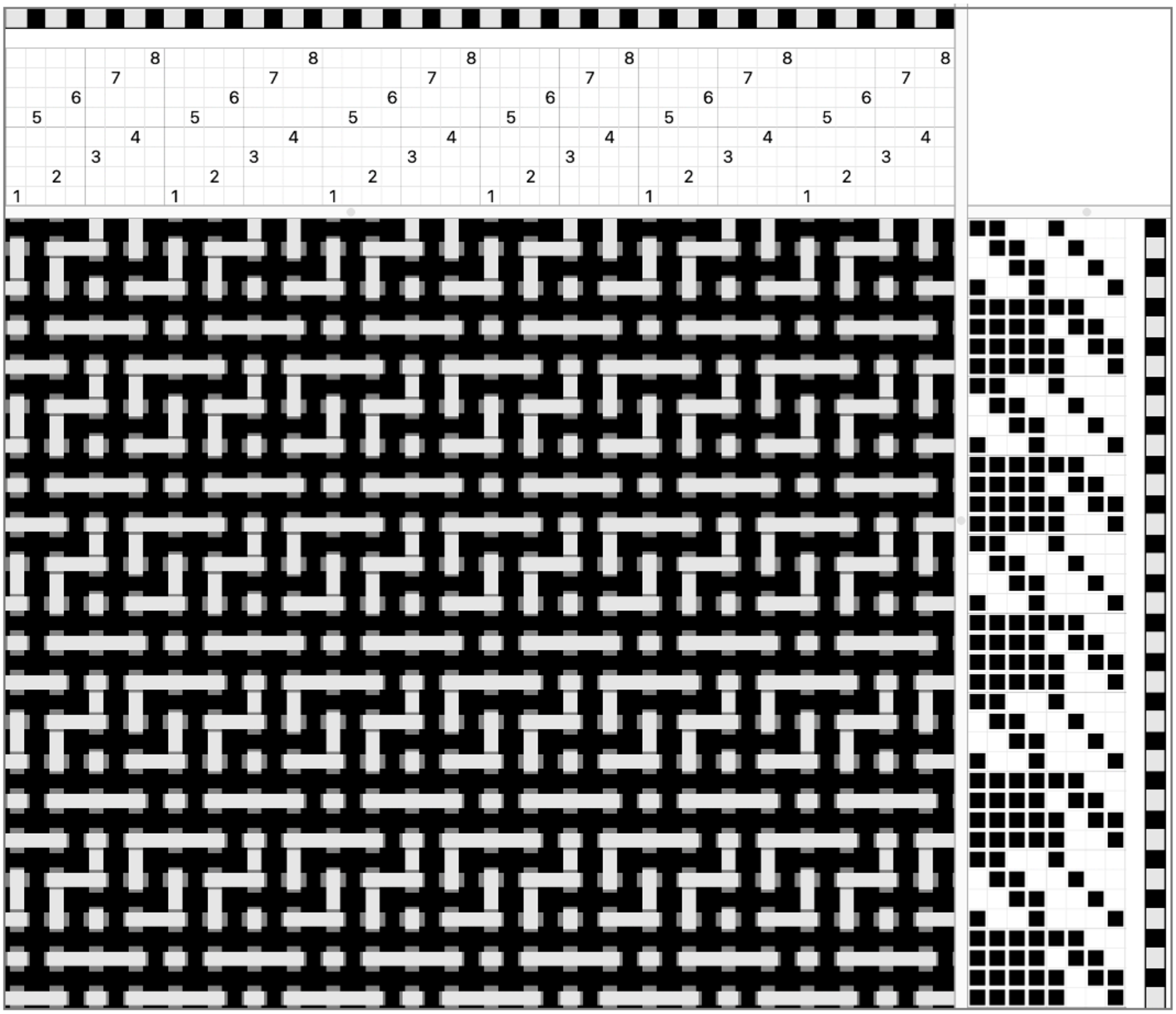

Supplementary Figure 15: Weave draft for one side of a double weave structure with tie downs.

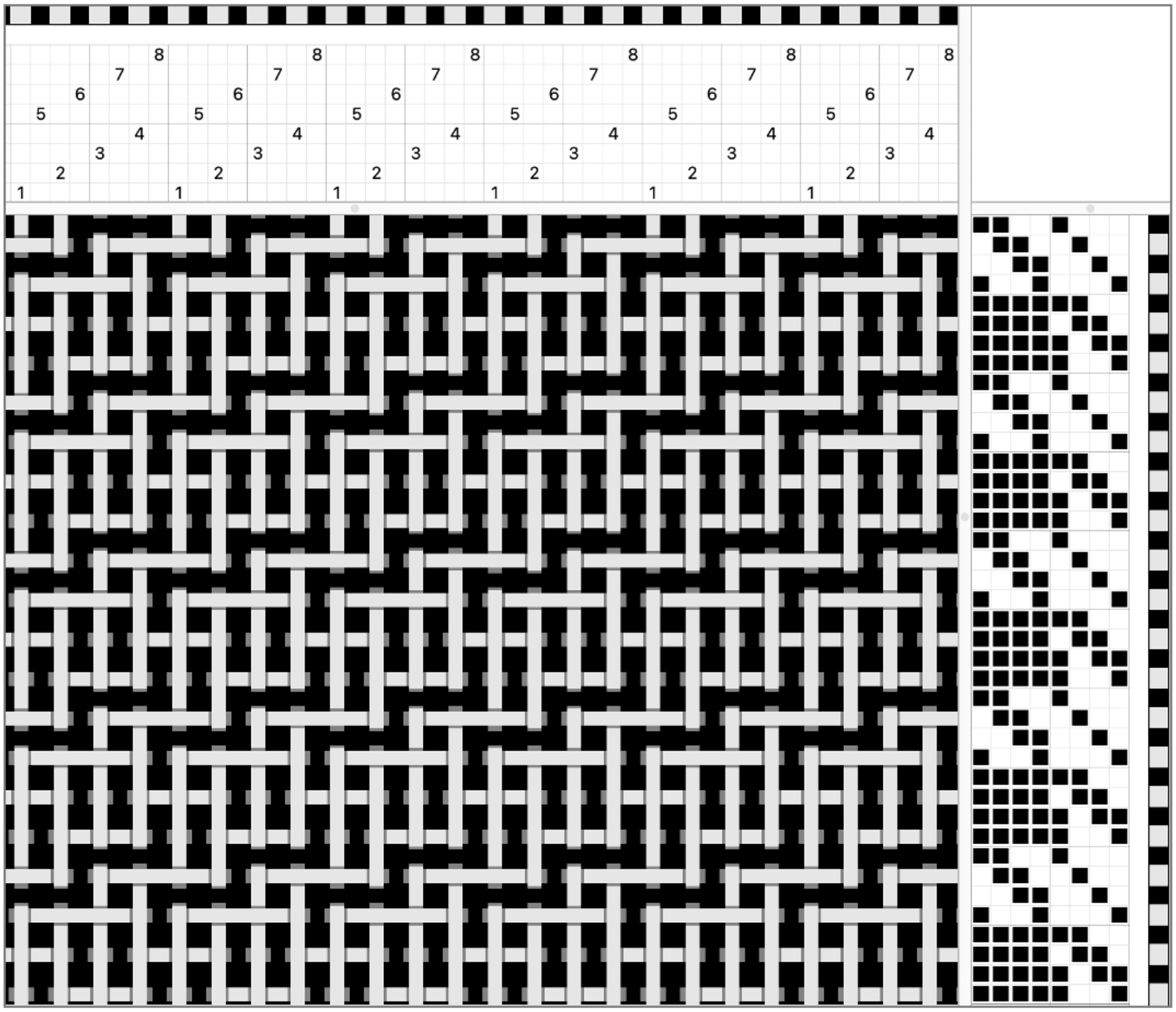

Supplementary Figure 16: Weave draft for the other side of a double weave structure with tie downs.

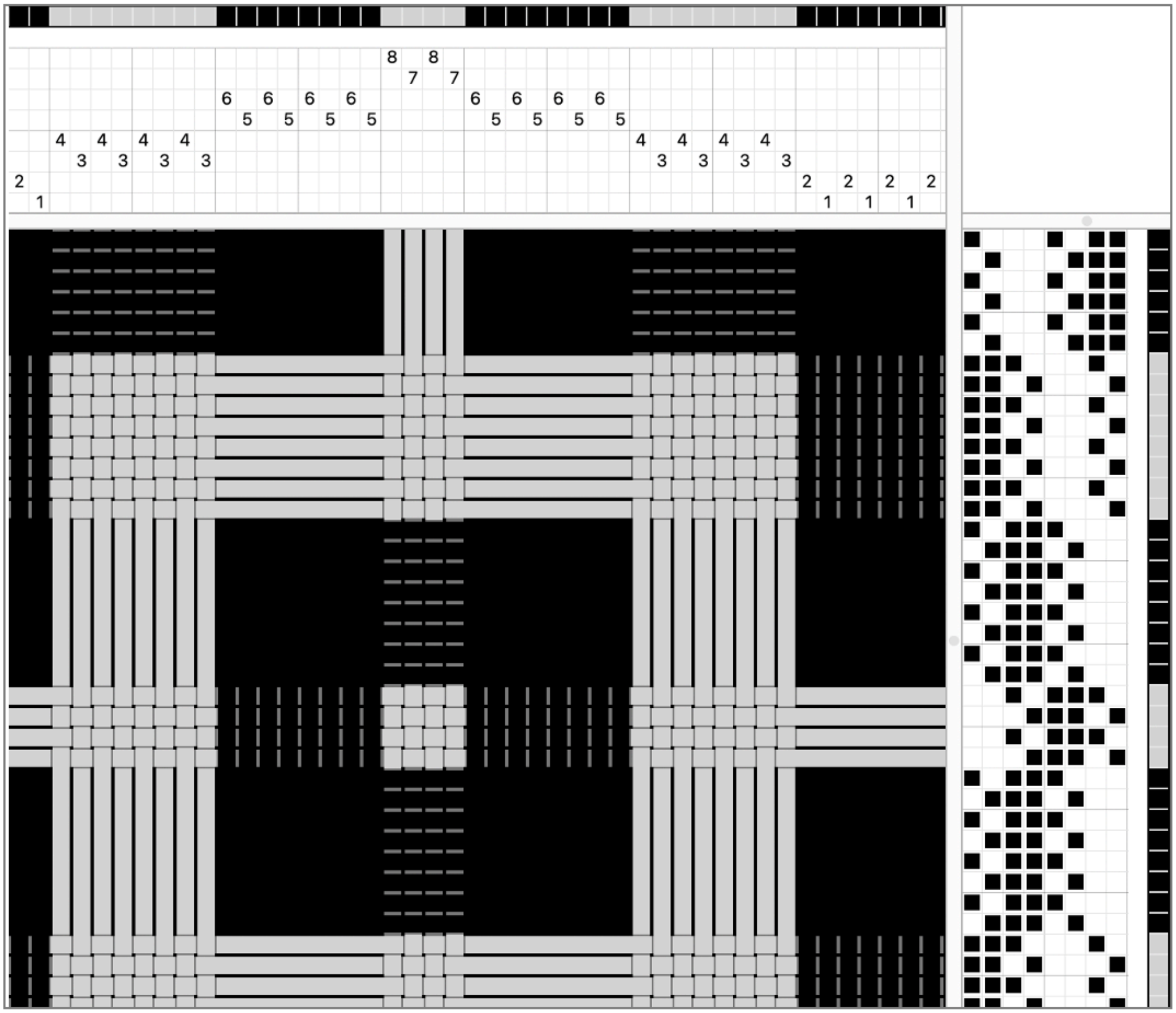

Supplementary Figure 17: Weave draft for one side of a double-deflected weft structure.

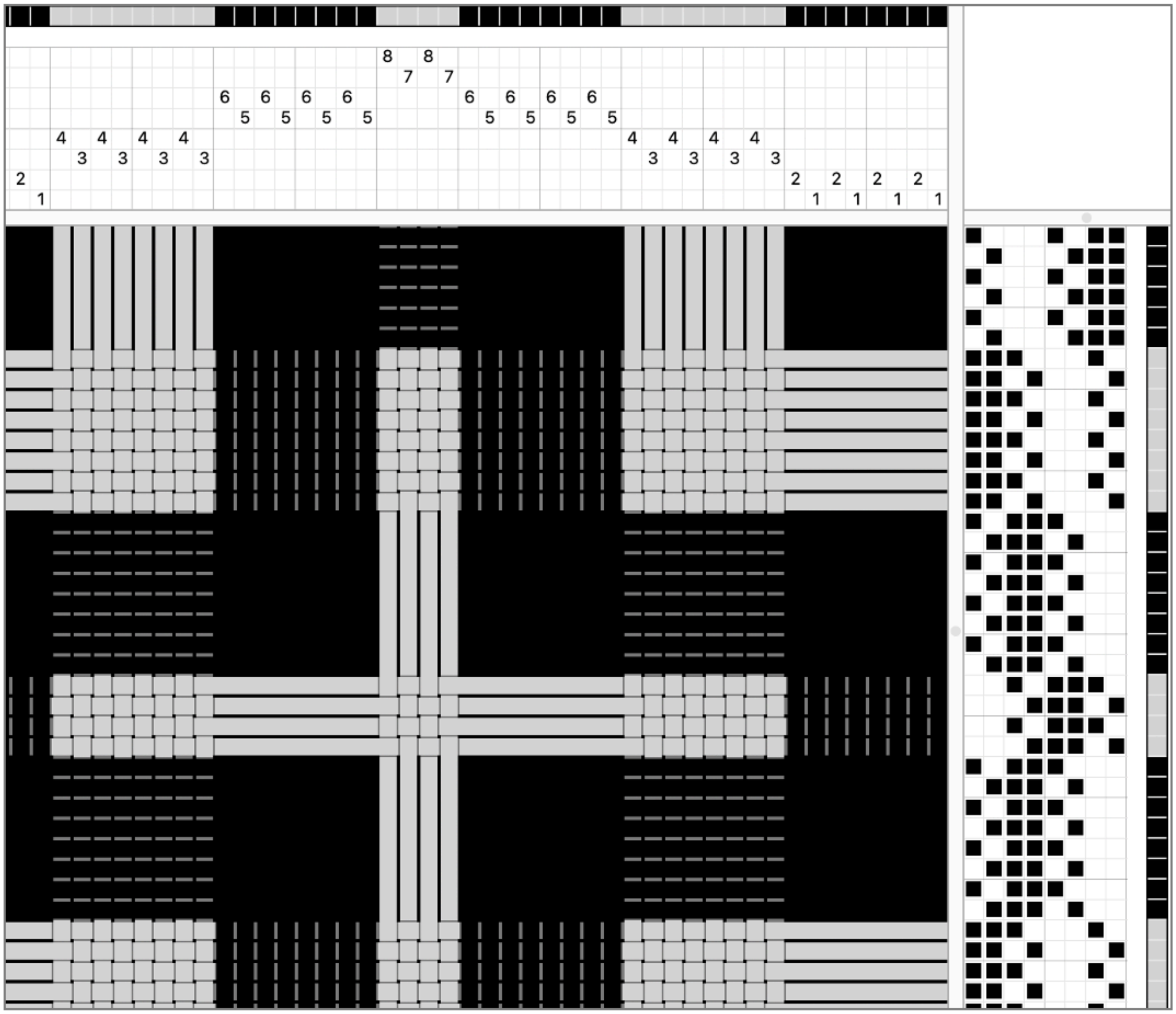

Supplementary Figure 18: Weave draft for the other side of a double-deflected weft structure.

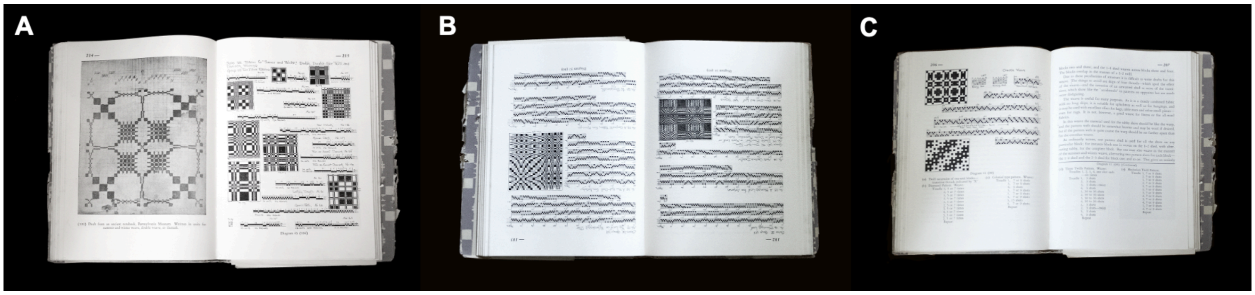

Supplementary Figure 19: Design inspiration for the fabricated garment (Figures 21 and 22) from Mary Atawater's *The Shuttle Craft Book of American Hand-Weaving* (first published in 1928). A) Overview of double woven structures, B) explanation of double woven structures, and C) additional weave structures with explanations.
